# Indole Diterpene Derivatives from the *Aspergillus flavus* GZWMJZ-288, an Endophytic Fungus from *Garcinia multiflora*

**DOI:** 10.3390/molecules28237931

**Published:** 2023-12-04

**Authors:** Dongyang Wang, Xiaohong Zhuang, Ying Yin, Dan Wu, Wenwen He, Weiming Zhu, Yanchao Xu, Mingxing Zuo, Liping Wang

**Affiliations:** 1 State Key Laboratory of Functions and Applications of Medicinal Plants, Guizhou Medical University, Guiyang 550014, China; wangdongyang@gmc.edu.cn (D.W.);; 2 Natural Products Research Center of Guizhou Province, Guiyang 550014, China; 3 School of Pharmaceutical Sciences, Guizhou Medical University, Guiyang 550025, China; 4 School of Medicine and Pharmacy, Ocean University of China, Qingdao 266003, China

**Keywords:** indole diterpene, endophytic fungus, secondary metabolite, *α*-glucosidase, NPC1L1

## Abstract

A new indole diterpene, 26-dihydroxyaflavininyl acetate (**1**), along with five known analogs (**2**–**6**) were isolated from the liquid fermentation of *Aspergillus flavus* GZWMJZ-288, an endophyte from *Garcinia multiflora*. The structures of these compounds were identified through NMR, MS, chemical reaction, and X-ray diffraction experiments. Enzyme inhibition activity screening found that compounds **1**, **4**, and **6** have a good binding affinity with NPC1L1, among which compound **6** exhibited a stronger binding ability than ezetimibe at a concentration of 10 µM. Moreover, compound **5** showed inhibitory activity against *α*-glucosidase with an IC_50_ value of 29.22 ± 0.83 µM, which is 13 times stronger than that of acarbose. The results suggest that these aflavinine analogs may serve as lead compounds for the development of drugs targeting NPC1L1 and *α*-glucosidase. The binding modes of the bioactive compounds with NPC1L1 and α-glucosidase were also performed through in silico docking studies.

## 1. Introduction

**Figure 1:**
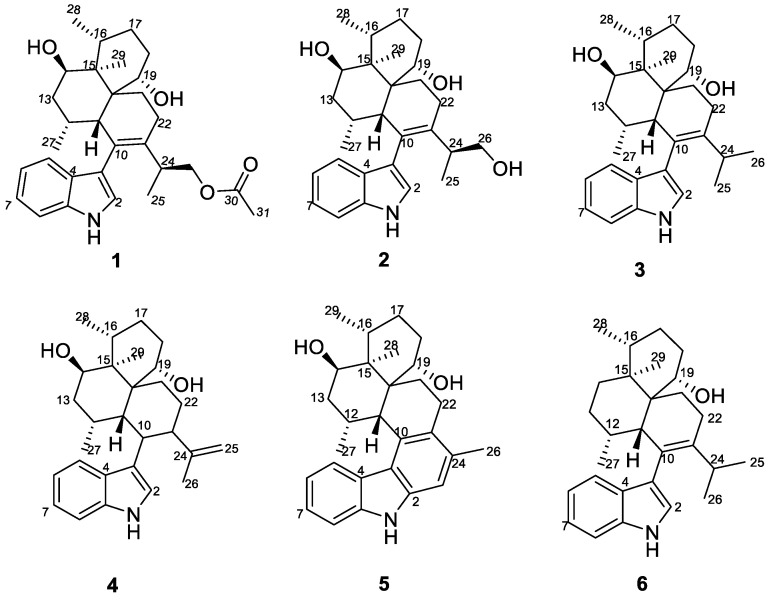
Structures of compounds **1**–**6**.

Endophytes and their host plants have formed close mutualism relationships in the process of long-term coevolution. Due to the unique biocatalytic mechanism and special living environment, endophytes can produce diverse natural products [1]. These compounds have a variety of biological activities, such as antimicrobial, anti-inflammatory, antioxidant, cytotoxic, and herbicides [2,3,4]. Previously, we have been committed to studying the secondary metabolites of endophytic fungi from *Garcinia multiflora*, and a series of new compounds with biological activities were found. Among them, a new kojic acid dimer was isolated from *Aspergillus flavus* GZWMJZ-288 during the rice solid fermentation process [5]. Furthermore, three new alkaloids were obtained through chemical transformation of the crude extract from this strain [6]. These results indicated that *Aspergillus flavus* GZWMJZ-288 has the potential for further research. Based on the OSMAC strategy, this fungus was fermented under liquid conditions, and the mycelium and liquid obtained from liquid fermentation were extracted using EtOAc and 80% acetone, respectively. The HPLC-DAD analysis spectra of both extracts show a series of chromatographic peaks with different types of UV absorption from the reported compounds from this strain. After chromatographic isolations, a new indole diterpene (**1**) along with five known compounds, dihydroxyaflavinin (**2**) [7], 14-hydroxyaflavinine (**3**) [8], 14-eoi-14-hydroxy10,23-dihydro-24,25-dehydroaflavinine (**4**) [9], aflavazole (**5**) [10], aflavinine (**6**) [11], were obtained. The structures of compounds **1**–**6** are shown in Figure 1. All of the isolated compounds contain similar skeletons to aflavinine, a class of the fascinating indole diterpene. The first aflavinine analog was isolated from *Aspergillus flavus* in 1980 [12]. Over the years, efforts have been made to explore its biological activities and applications. However, only 18 natural aflavinine analogs have been discovered, and biological studies have focused on its anti-insect, antiviral, and anticancer activities [7,12,13,14,15,16,17]. In this study, we aim to explore whether these compounds can serve as enzyme inhibitors.

Hypercholesterolemia is a significant risk factor for cardiovascular diseases. Studies have revealed that Niemann-Pick C1-Like 1 (NPC1L1) is a multipass membrane protein that mediates the intestinal absorption of cholesterol [18]. The inhibition of NPC1L1 can significantly reduce the level of serum cholesterol. However, there is currently a lack of highly effective NPC1L1 inhibitors, with only one approved NPC1L1 inhibitor, ezetimibe. The number of highly effective NPC1L1 inhibitors remains low, mainly including orlistat, peptides, and phenolic compounds [19]. Therefore, searching for NPC1L1 inhibitors is still critical for developing new cholesterol-lowering medicines and treating cardiovascular diseases. On the other hand, it has been noticed that NPC1L1 inhibitors from microbial sources have not been reported.

In this study, the combining potential of compounds **1**, **4**, and **6** with NPC1L1 was assessed. The results revealed that all three compounds bind well with NPC1L1, and compound **6** exhibits a stronger binding ability than ezetimibe. Additionally, the inhibitory effect of the isolated compounds on α-glucosidase was also evaluated, with compound 5 exhibiting significant inhibitory activity. This article reports on the isolation, structural elucidation, and biological activities of these compounds. To the best of our knowledge, the abilities of aflavinines to inhibit α-glucosidase and bind with the cholesterol transporter NPC1L1 have never been reported before.

## 2. Results and Discussion

### 2.1. Strain Fermentation and Secondary Metabolites’ Isolation

A total of 18 L of fermentation broth of *Aspergillus flavus* GZWMJZ-288 was obtained. The sediment and liquid were separated and extracted using EtOAc and 80% aqueous acetone, respectively. After concentration under vacuum, the EtOAc layer yielded 20.8 g of extract and the acetone layer yielded 7.4 g of extract. Subsequently, both extracts were successively subjected to multiple chromatographic separations. Finally, compounds **1**–**4** and **6** were obtained from the EtOAc extract with a mass of 10.2 mg, 10.5 mg, 9.6 mg, 10.2 mg, and 16.2 mg, respectively. The acetone extract yielded 5.6 mg of compound **5**.

### 2.2. Structural Characterization of Isolated Compounds

**Figure 2:**
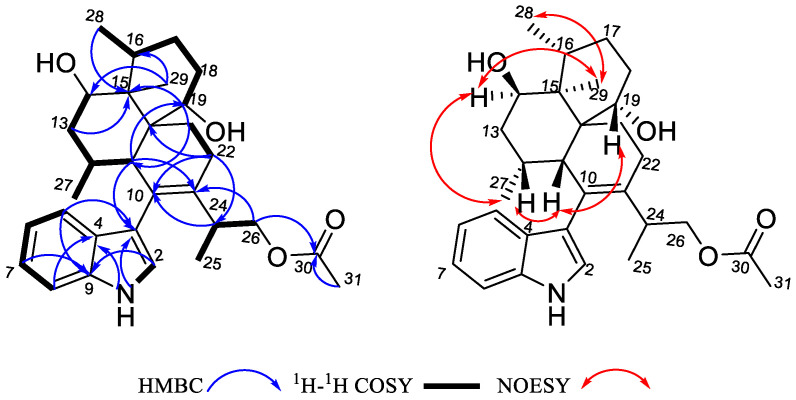
The key 2D NMR correlations of compound **1**.

**Figure 3:**
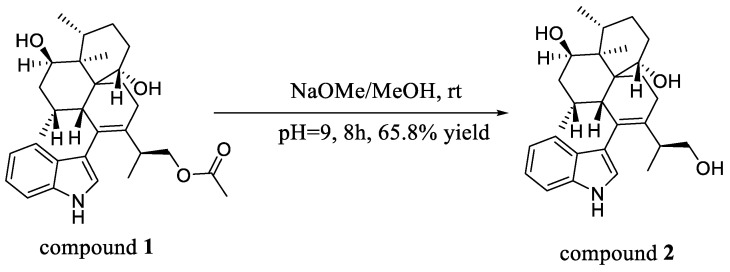
Hydrolysis reaction of compound **1**.

**Figure 4:**
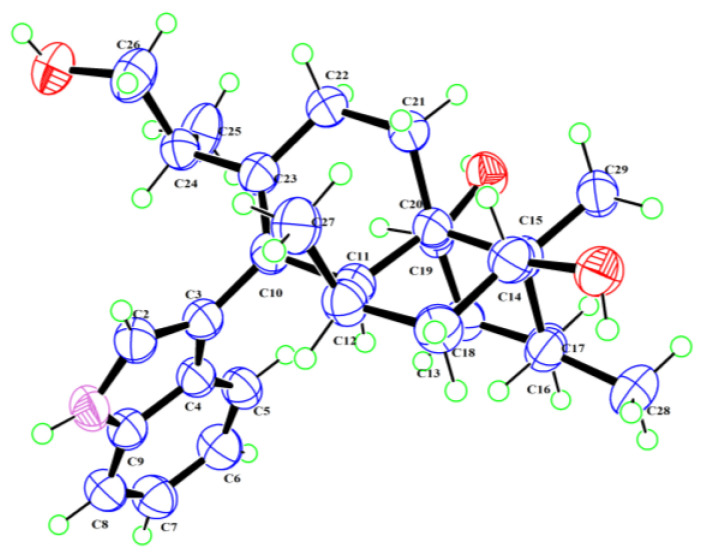
The X-ray single-crystal diffraction of **2**. Carbon, hydrogen, nitrogen, and oxygen atoms are colored blue, green, purple, and red, respectively.

26-dihydroxyaflavininyl acetate (**1**) was obtained as a yellow powder. The molecular formula was deduced as C_30_H_41_NO_4_ based on the HRESIMS ion peak at *m*/*z* 502.29172 [M + Na]^+^ (calcd. for C_30_H_41_NO_4_Na = 502.29278), with eleven degrees of unsaturation. Its IR (KBr) spectrum exhibited absorptions at 3373 cm^−1^ (hydroxy) and 1658/1243/1094 cm^−1^ (ester group). The NMR spectra showed five sp^2^-quaternary carbons’ signals, five sp^2^-methines carbons’ signals, two sp^3^-quaternary carbons’ signals, six sp^3^-methine carbons’ signals (including two oxygenated methines), six sp^3^-methylene carbons’ signals (including one oxygenated methines), and four methyl signals (Table 1). These data were very close to those of compound **2** [14]. The ^1^H-^1^H COSY correlation between H-5 (*δ*_H_ 7.29)/H-6 (*δ*_H_ 6.95)/H-7 (*δ*_H_ 7.05)/H-8 (*δ*_H_ 7.35), and NH (*δ*_H_ 10.93)/H-2 (*δ*_H_ 6.93) (Figure 2 and Figure S6), and the key HMBC correlations from NH to C-3 (*δ*_C_ 116.1), NH and H-8 to C-4 (*δ*_C_ 126.8), and H-5 to C-9 (*δ*_C_ 135.8) indicated the presence of an indole part. And the presence of three six-membered rings is also evidenced by H-11 (*δ*_H_ 2.34) to C-19 (*δ*_C_ 68.0)/C-23 (*δ*_C_ 135.6), H-12 (*δ*_H_ 1.98) to C-14 (*δ*_C_ 69.5), H-21 (*δ*_H_ 1.63) to C-15 (*δ*_C_ 42.9)/C-11 (*δ*_C_ 40.0), and H-22 ((*δ*_H_ 1.95) to C-10 (*δ*_C_ 128.9)/C-20 (*δ*_C_ 43.9) in the HMBC spectrum of compound **1**. The HMBC correlation from H-11 to C-3 confirmed that these two structures are connected through C-3 and C-10, suggesting that compound **1** has a similar skeleton to compound **2**. Especially, correlations between H-26 (*δ*_H_ 3.84, 3.93) and C-30 (*δ*_C_ 170.2), H-31 (*δ*_H_ 1.98), and C-30 were observed in the HMBC spectrum (Figure 2 and Figure S5) of **1**, indicating that compound **1** has one more acetyl group than compound **2**. So, the plane structure of compound **1** is determined as shown in Figure 1. The correlations of H-27 (*δ*_H_ 1.11) with H-14 (*δ*_H_ 3.81), H-29 with H-28 (*δ*_H_ 0.93) and H-14, as well as H-11 with H-12 and H-19 (*δ*_H_ 4.27) were detected through NOESY (Figure 2 and Figure S7); thus, the relative configurations of C-11, C-12, C-14, C-15, C-16, and C-19 in compound **1** were determined to be the same as the corresponding positions in compound **2**. To determine whether the absolute configuration of compound **1** is the same as that of compound **2**, the acetyl group in compound **1** was hydrolyzed using NaOMe (Figure 3). The ^1^H NMR spectra of the hydrolysate of compound **1** (Table 1, Figure S18) matched those of compound **2** and their optical rotation data were also consistent (hydrolysate of **1**: [*α*${]}_{D}^{24}$ + 80.1, **2**: [*α*${]}_{D}^{24}$ + 73.3), which indicated that **1** and **2** shared the same stereochemical configuration. In addition, the absolute configuration of compound **2** was determined using single-crystal X-ray diffraction (Figure 4, CCDC: 2222865). Thus, the absolute configuration of compound **1** was finally confirmed as 11*S*, 12*R*, 14*R*, 15*S*, 16*R*, 19*S*, 20*S*, and 24*S*.

### 2.3. Biological Activities

#### 2.3.1. Results of Combining Ability with NPC1L1

**Figure 5:**
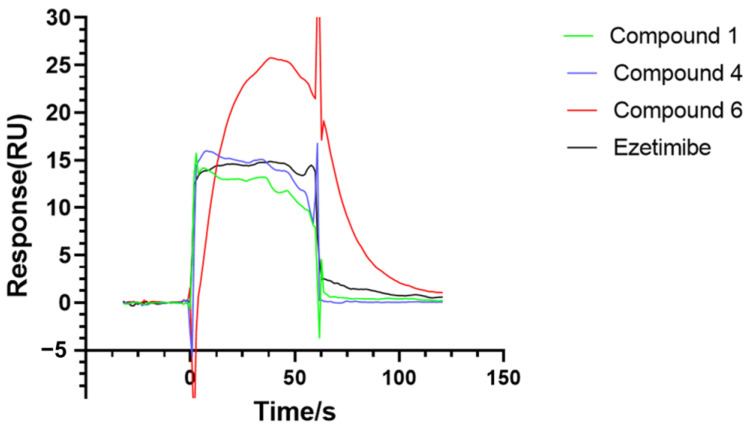
Based on the SPR principle, the Biacore instrument was used to analyze the interaction (ezetimibe, compounds **1**, **4**, **6**) with NPC1L1.

**Figure 6:**
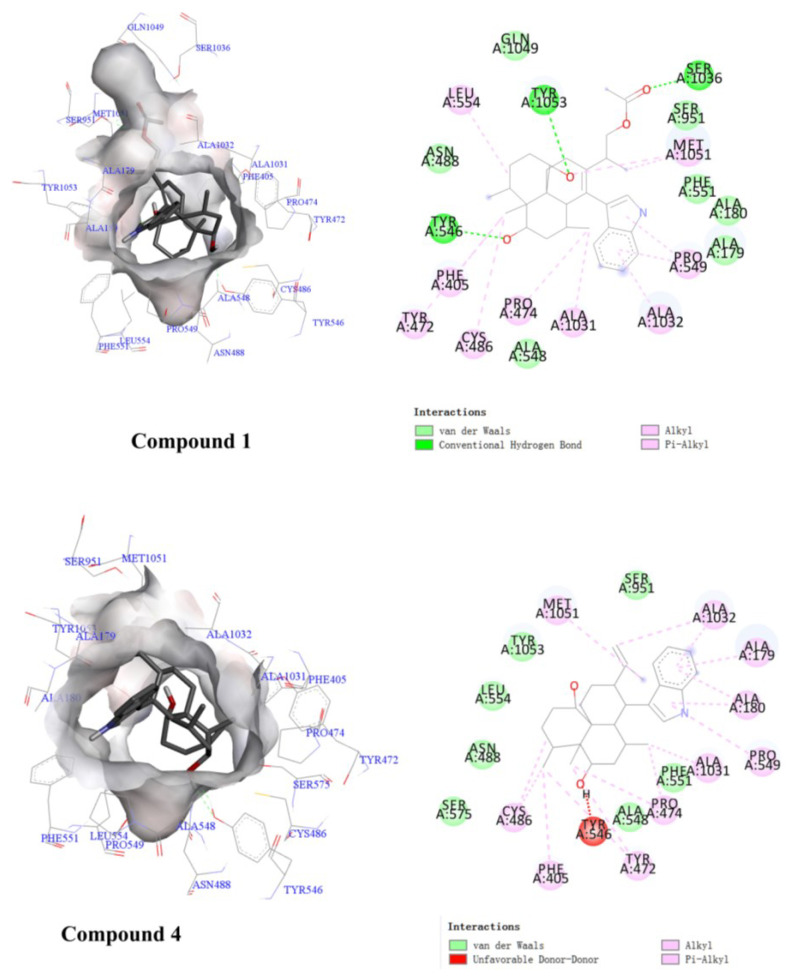

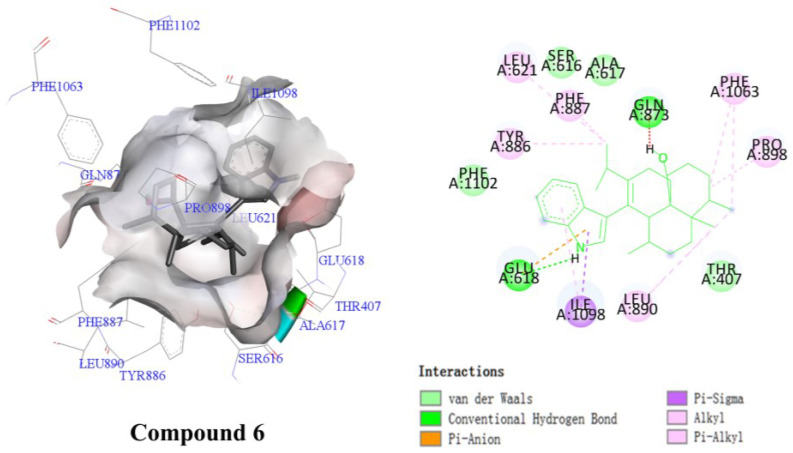
Ligand interaction binding pose and diagram of compounds **1**, **4**, and **6** with the NPC1L1 (PDB: 6V3H).

Surface Plasmon Resonance (SPR) can be used to characterize the interaction between target proteins and the compounds [19,20,21]. The results showed that compounds **1**, **4**, and **6** could bind well with NPC1L1, and the binding ability of **6** to NPC1L1 was stronger than that of ezetimibe at a concentration of 10 μM (Figure 5). To further investigate the bonding poses of these three compounds with NPC1L1, molecular docking studies were carried out using Autodock Vina with the crystal structure of the cholesterol transporter (PDB ID: 6V3H). The results indicated that compound **1** could interact with the residues of Leu554, Tyr1053, Ser1036, Met1051, Pro549, Ala1032, Ala1031, Pro474, Cys486, Tyr472, and Tyr546 (binding energy: −8.8 kcal/mol). Compound **4** could interact with the residues of Met1051, Ala1032, Ala179, Ala180, Pro549, Ala1031, Pro474, Tyr472, Tyr546, Phe405, and Cys486 (binding energy: −8.8 kcal/mol), and compound **6** showed interactions with amino acid residues Phe1063, Pro898, Lfu890, Ilf1098, Glu618, Tyr886, Leu621, Phe887, and Gin873 in the NPC1L1 (binding energy: −9.4 kcal/mol) (Figure 6). These results serve as a reference for subsequent structural modification efforts to enhance their binding ability with NPC1L1.

#### 2.3.2. α-Glucosidase Inhibitory Activity

**Figure 7:**
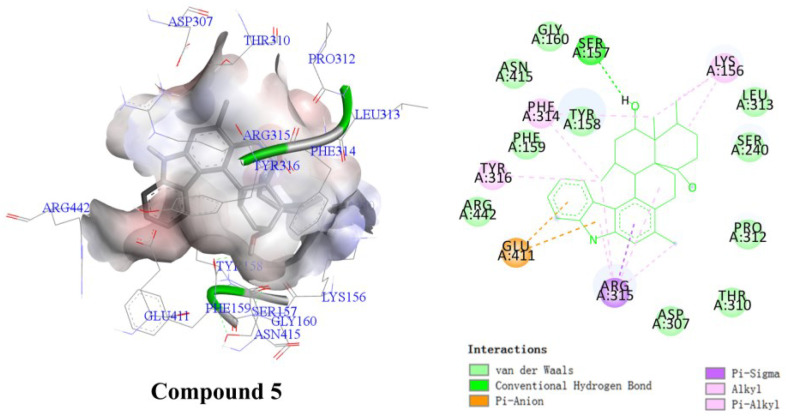
Ligand interaction binding pose and diagram of **5** at the active side of isomaltase.

The *α*-glucosidase inhibitory activity of **1**–**6** was preliminarily investigated. The results showed that compound **5** exhibited inhibitory activity against the *α*-glucosidase from *Saccharomyces cerevisiae* with an IC_50_ value of 29.22 ± 0.83 μM, while the IC_50_ value of acarbose was 387.27 ± 19.02 μM (Table 2). To investigate the binding modes of compound **5** with *α*-glucosidase, molecular docking studies were carried out using Autodock Vina with the crystal structure of isomaltase from *Saccharomyces cerevisiae* (PDB ID: 3A4A) [22,23]. According to the docking results, compound **5** could generate significant interactions with the amino acid residues Lys156, Arg315, Glu411, Arg442, Tyr316, Phe314, Tyr158, and Ser157 in the isomaltose (Figure 7), respectively.

## 3. Materials and Methods

### 3.1. General Experimental Procedures

Using TMS as an internal standard, ^1^H NMR, ^13^C NMR, and 2D NMR spectra were recorded on Bruker Advance NEO 600 spectrometer (Bruker, Billerica, MA, USA). IR spectra were measured on an iCAN 9 infrared spectrophotometer (Tianjin Nengpu Technology Co., Ltd, Tianjin, China) with KBr disks. UV spectra were detected on a Cary 60-UV-Vis spectrometer (Agilent, Santa Clara, CA, USA). ESI-MS was tested on an Agilent 1100 instrument mass spectrometer (Agilent, Santa Clara, CA, USA), HR-ESI-MS analysis and testing were performed on a Thermo ultimate 3000/Q Exactive focus instrument mass spectrometer (Thermo Fisher Scientific, Waltham, MA, USA). The optical rotations were measured on a Rudolph Autopol1 automatic polarimeter (Rudolph Research Analytical, Hackettstown, NJ, USA). The X-ray data were obtained using the Bruker Smart 1000 CCD area detector diffractometer with graphite monochromatic Cu-Kα radiation. Column chromatography was performed using silica gel (100–200 and 200–300 mesh) purchased from Qingdao Puke Parting Materials Co., Ltd., Qingdao, China. SepaBean machine was equipped with SepaFlash columns (Santai Technologies Inc., Changzhou, China). Gel permeation chromatography was performed using a Sephadex LH-20 column from Amersham Biosciences, Uppsala, Sweden, and a Toyopearl HW-40F column from Tosoh Bioscience, Tokyo, Japan. HPLC-DAD analyses were performed on a Hitachi Primide machine equipped with a 1430 Diode Array Detector, an 1110 pump, and a YMC-pack ODS-A column (5 μm, 4.6 × 150 mm, 1 mL/min). An ODS column on the same Hitachi Primide machine (YMC-pack ODS-A, 5 μm, 10 × 250 mm, 4 mL/min, Tokyo, Japan) was used for semi-preparative HPLC separation. The absorbance at 405 nm was recorded using the Varioskan LUX multimode microplate reader (Thermo Fisher Scientific™, Waltham, MA, USA).

### 3.2. Strain Isolation and Fermentation

The strain GZWMJZ-288 was isolated from the fruit of *Garcinia multiflora* and identified by the phylogenetic trees of the ITS region sequence of the fungus, which has been submitted to GenBank (accession number No. MH041190) [5,6]. The strain was grown on PDA plates at 28 °C for 4 days. After that, the fungus was cut into small pieces and cultivated under static conditions at 28 °C for 60 days in 60 × 1000 mL conical flasks containing liquid medium (300 mL/flask) composed of water-soluble starch (1%) and peptone (0.1%).

### 3.3. Extraction and Isolation

Fermentation was terminated with a small amount of EtOAc, and then the culture was filtered with gauze and divided into two parts, the sediment and fermentation broth, extracted with EtOAc and 80% acetone, respectively. Finally, 20.8 g of concentrated extract of the EtOAc layer and 7.4 g of acetone layer concentrate were obtained.

The combined EtOAc extract (20.8 g) was separated using silica gel chromatographed using PE-EtOAc (*v*/*v*, 100:0, 50:1, 25:1, 10:1, 5:1, 3:1, 1:1) and DCM-EtOAc (*v*/*v*, 20:1, 10:1, 5:1, 3:1, 1:1) as eluents, resulting in 28 fractions. Fraction (Fr.) 9 (176.0 mg) was combined with the SepaBean machine and purified through semi-preparative HPLC (68% ACN/H_2_O with 0.05 trifluoroacetic acid (TFA), 4 mL/min) on an ODS column to obtain compound **1** (10.2 mg, *t*_R_ 5.6 min). Fr. 8 (211.0 mg) was chromatographed on a silica gel column using a step gradient elution to obtain four subfractions (Fr. 8.1~Fr. 8.4). Fr. 8.3 (89.5 mg) was purified through semi-preparative HPLC (70% MeOH/H_2_O with 0.05% TFA, 4 mL/min) on an ODS column to afford compound **2** (10.5 mg, *t*_R_ 7.2 min). Fr. 16 (58.2 mg) was purified through semi-preparative HPLC (80% MeOH/H_2_O with 0.05% TFA, 4 mL/min) on an ODS column to acquire compound **3** (9.6 mg, *t*_R_ 10.5 min). A reverse silica gel column was used for Fr. 9 (297.0 mg) to obtain five components. Fr. 9.4 was separated using an HW-40F column with 50% DCM–MeOH, and purified through semi-preparative HPLC (80% MeOH/H_2_O with 0.05 TFA, 4 mL/min) on an ODS column to receive compound **4** (10.2 mg, *t*_R_ 10.1 min) and compound **6** (16.2 mg, *t*_R_ 12.5 min). The acetone extract (7.4 g) was isolated using a Sephadex LH-20 column with 50% DCM–MeOH into 13 fractions. Fr. 11 (89.0 mg) was purified through semi-preparative HPLC (55% ACN/H_2_O with 0.05% TFA, 4 mL/min) on an ODS column to obtain compound **5** (5.6 mg, *t*_R_ 8.9 min).

### 3.4. Physical Properties and Spectral Data of ***1***–***6***

Compound **1**: yellow powder; ECD (2.1 *m*M, MeOH) *λ*max (Δ*ε*) 231 (+8.6), 282 (+0.83), 291 (+0.80) nm; [*α*${]}_{D}^{24}$ + 85.7 (*c* 0.56, MeOH); UV (MeOH) λ_max_ (log *ε*) 223 (3.8), 282 (3.0), 291 (2.9) nm; IR (KBr) *ν*_max_ 3373, 2929, 1658, 1458, 1379, 1342, 1243, 1094, 1024, 1018, 742 cm^−1^; for ^1^H NMR and ^13^C NMR data, see Table 1 and Figures S2–S7; HRESIMS *m*/*z* 502.29172 [M + Na]^+^ (Figure S1), molecular formula: C_30_H_41_NO_4_.

Compound **2**: light yellow crystals. The molecular formula is C_28_H_39_NO_3_ (*m*/*z* 436.2 [M − H]^−^) determined using ESIMS. [*α*${]}_{D}^{24}$ + 73.3 (*c* 0.04, MeOH). The ^1^H NMR and ^13^C NMR data (Table 1, Figures S8 and S9) proved that compound **2** was dihydroxyaflavinin [7,14].

Crystal data for compound **2** (Tables S1–S3). C_28_H_39_O_3_N, *M* = 437.60 g/mol, monoclinic, space group *P* 2_1_, *a* = 9.3756 (3) Å, *b* = 10.4260 (4) Å, *c* = 12.5667 (5) Å, *α* = 90°, *β* = 96.673° (2), *γ* = 90°, *V* = 1220.07 (8) Å^3^, *T* = 150 K, space group *P*1 21 1, *Z* = 2, *μ* (Cu Kα) = 0.595 mm^−1^, *D_calc_* = 1.191 g/cm^3^, crystal dimensions 0.2 × 0.15 × 0.1 mm, *μ* = 0.595 mm^−1^, *F* (000) = 476.0. 10,667 reflections measured (7.082° ≤ 2θ ≤ 144.774°), 4676 independent reflections (*R_int_* = 0.0407, *R_sigma_* = 0.0547). The final *R*_1_ values were 0.0710 (*I* > 2*σ* (*I*)). The final *wR* (*F*^2^) values were 0.1607 (*I* > 2*σ* (*I*)). The final *R*_1_ values were 0.0727 (all data). The final *wR*(*F*^2^) values were 0.1633 (all data). The goodness of fit on *F*^2^ was 1.087. Flack parameter = 0.06 (13). CCDC: 2222865.

Compound **3**: light yellow solid. The molecular formula is C_28_H_39_NO_2_ (*m*/*z* 444.2 [M + Na]^+^) confirmed using ESIMS. [*α*${]}_{D}^{24}$ + 13.8 (*c* 0.29, MeOH). The ^1^H NMR and ^13^C NMR data (Table S4, Figures S10 and S11) indicated that compound **3** was 14-hydroxyaflavinine [8].

Compound **4**: yellow solid. The molecular formula is C_28_H_39_NO_2_ (*m*/*z* 420.2 [M − H]^−^) determined using ESIMS. [*α*${]}_{D}^{24}$ + 6.1 (*c* 0.66, MeOH). The ^1^H NMR and ^13^C NMR data (Table S4, Figures S12 and S13) proved that compound **4** was monhydroxyisoaflavinine [9,14].

Compound **5**: yellow powder. The molecular formula is C_28_H_35_NO_2_ (*m*/*z* 440.4 [M + Na]^+^) determined using ESIMS. [*α*${]}_{D}^{24}$ + 17.0 (*c* 0.47, MeOH). The ^1^H NMR and ^13^C NMR data (Table S4, Figures S14 and S15) proved that compound **5** was aflavazole [10,24].

Compound **6**: light yellow solid. The molecular formula is C_28_H_39_NO (*m*/*z* 444.5 [M + K]^+^) determined using ESIMS. [*α*${]}_{D}^{24}$ + 59.6 (*c* 1.14, MeOH). The ^1^H NMR and ^13^C NMR data (Table S4, Figures S16 and S17) proved that compound **6** was aflavinine [11].

### 3.5. Hydrolysis Reaction of Compound ***1***

Compound **1** (5 mg, 0.01 mmol) was added to a solution of NaOMe in methanol (pH 9.0, 2 mL). The mixture was stirred at room temperature for 8 h, and the pH was adjusted to 4.0 with an aqueous HCl solution (0.1 mol/L). Then, the solvent was evaporated to dryness under vacuum, and the solid was suspended in water (5 mL). The suspension was extracted three times with 5 mL of EtOAc, and the combined organic phase was dried over Na_2_SO_4_ and removed in vacuo to yield a paste residue. Finally, the residue was purified through semi-preparative HPLC (70% MeOH/H_2_O with 0.05% TFA, 4 mL/min) on an ODS column to afford the hydrolysate of **1** (3.0 mg, *t*_R_ 7.2 min). [*α*${]}_{D}^{24}$ + 80.1 (*c* 0.04, MeOH), ESIMS *m*/*z* 436.2 [M − H]^−^, ^1^H-NMR (600 MHz, DMSO-*d*_6_): see Table 1 and Figure S18.

### 3.6. Combining Ability Test with NPC1L1 

NPC1L1 is currently considered to be the key protein for cholesterol absorption [25]. It is impossible to evaluate the activity of targeting compounds on NPC1L1 through colorimetry assay due to NPC1L1′s lack of catalytic activity. The experiment was carried out under fixed concentration at 10 μM with ezetimibe as a positive control. Excess reactive esters on the sensor chip surface were blocked with 1 M ethanolamine (pH 8.5) for 7 min. The flow cell used for reference was activated and blocked as described above but remained uncoupled. Binding is typically reported in response units (RUs), which is defined as the response obtained from the flow cell containing the immobilized receptor minus the response obtained from the reference flow cell [19,20,21]. CM5 series sensor chips and coupling reagents EDC and NHS were purchased from GE. The surface of the flow cell was activated for 7 min using a 1:1 mixture of 100 mM EDC and 100 mM NHS at a flow rate of 10 μL/mi. Subsequently, the corresponding proteins were injected over the surface for 7 min using the time and flow method (final immobilization level: 15,000 RU). Excess reactive esters on the sensor chip surface were blocked with 1 M ethanolamine (pH 8.5) for 7 min. The ability of compounds to combine with NPC1L1 protein was determined by the response value.

### 3.7. α-Glucosidase Inhibitory Activity

The inhibitory activity of *α*-glucosidase was determined using the reported method [5,6]. All of the six compounds were dissolved in 20% DMSO, and the other reagents were dissolved in phosphate-buffered saline (0.1 M PBS, pH 6.8). An amount of 20 μL of acarbose (2.5 mg/mL, Sigma, Ronkonkoma, NY, USA) was added as a positive control group and 20 μL of α-glucosidase (0.25 U/mL) was added to sample solutions in 96-well plates and preincubated at 37 °C for 15 min. Then, 20 μL of PNPG (4-nitrophenyl-α-D-glucopyranoside, 2.5 mM, Macklin, Belfast, Northern Ireland) was added to each well of the 96-well microplate and incubated at 37 °C for 30 min. Finally, 80 μL of Na_2_CO_3_ solution (0.2 M) was added to each well to terminate the reaction. All experiments were repeated three times and the absorbance was measured at 405 nm.

### 3.8. Details of Docking Research

The docking studies of compounds in the active site of *α*-glucosidase and NPC1L1 protein were carried out using AutoDock Vina on PyRx version 0.8 [6,22,23]. The 3D structures of the compounds were obtained using ChemBioDraw 15.0 and ChemBio3D 15.0 software. The Auto DockTools 1.5.6 package was employed to generate the docking input files. The crystal structure of *α*-glucosidase from *Saccharomyces cerevisiae* co-crystallized with glucose (PDB ID: 3A4A) was used as the target *α*-glucosidase proteins [26], and the crystal structure of NPC1L1 protein in complex with an ezetimibe analog (PDB ID: 6V3H) was used as the receptor of cholesterol transporter. The protein file (PDB) was further optimized by removing water and ligand molecules and adding hydrogen atoms. The search grids of α-glucosidases (PDB ID: 3A4A) were respectively identified as center _x: 21.301, _y: −0.8274, and _z: 14.8961 with a grid box of 60 × 60 × 60, while the search grids of NPC1L1 protein was identified as center _x: 192.916, _y: 121.116, and _z: 130.298 with a grid box of 80 × 80 × 80. The number of runs was set at 100 in the search parameter. The best-scoring pose as judged by the Vina docking score was chosen. The binding orientations, molecular modeling, and evaluation of the hydrogen bonds and other interactions were determined using Discovery Studio Visualizer (Accelrys, Cambridge, UK) [27]. As positive controls for our docking studies, the glucose and ezetimibe analog split from PDB files of 3A4A and 6V3H were re-docked with their corresponding receptors. The binding poses of these two ligands were in agreement with their experimentally determined structures in the corresponding complexes, and these results validated the soundness of our docking methodology.

## 4. Conclusions

In this study, six indole diterpenoids containing both aflavinine and indole rings were isolated and identified from the fermentation extract of the endophytic fungus *Aspergillus* sp. GZWMJZ-288 based on the OSMAC strategy. Among them, one new 26-dihydroxyaflavininyl acetate (**1**) was discovered, and NMR, MS, chemical reaction, and X-ray diffraction experiments were used to determine its structure. Compound **6** showed a stronger binding ability with NPC1L1 than ezetimibe (the only approved NPC1L1 inhibitor drug), while compounds **1** and **4** displayed comparable binding ability with NPC1L1. These three compounds represent a new class of NPC1L1 inhibitors that differ from previously reported structures, providing novel leads for the development of future NPC1L1-targeted drugs. Meanwhile, the inhibitory activities of the isolated compounds against *α*-glucosidase were also assayed. Compound **5** exhibited a strong α-glucosidase inhibitory activity, with an IC_50_ value that was 13 times stronger than that of acarbose, making it a promising candidate for the development of new anti-diabetic drugs.

## Figures and Tables

**Table 1:** ^1^H (600 MHz) and ^13^C (150 MHz) NMR data of **1**, **2** and hydrolysate of **1** in DMSO-*d*_6_.

Position	1		2		Hydrolysate of 1
	*δ*_C_	*δ*_H_, (*J* in Hz)	*δ*_C_	*δ*_H_, (*J* in Hz)	*δ*_H_, (*J* in Hz)
2	122.3, CH	6.93, d (2.0)	122.5, CH	6.98, d (2.0)	6.97, d (2.1)
3	116.1, C		118.4, C		
4	126.8, C		127.4, C		
5	118.4, CH	7.29, d (7.9)	118.7, CH	7.35, d (8.1)	7.34, d (7.8)
6	118.5, CH	6.95, t (7.9)	116.4, CH	6.94, t (8.1)	6.93, t (7.8)
7	120.8, CH	7.05, t (7.6)	120.7, CH	7.04, t (7.7)	7.04, t (7.5)
8	111.6, CH	7.35, d (7.6)	111.5, CH	7.34, d (7.7)	7.32, d (7.5)
9	135.8, C		135.9, C		
10	128.9, C		126.9, C		
11	43.2, CH	2.34, d (5.6)	43.2, CH	2.35, d (6.3)	2.33, d (5.9)
12	29.5, CH	1.96–2.00, overlap	29.6, CH	1.92–2.02, overlap	1.92–2.01, overlap
13	35.3, CH_2_	1.36, d (12.3) 1.76–1.81, m	35.4, CH_2_	1.35, d (12.2) 1.79, ddd (12.2, 12.2, 5.9)	1.34, d (12.5) 1.78, ddd (12.5, 12.5, 5.7)
14	69.5, CH	3.80–3.83, overlap	68.1, CH	3.83, ddd (11.9, 4.3, 3.7)	3.81, ddd (12.5, 4.5 3.8)
15	42.9, C		43.9, C		
16	31.0, CH	1.96–2.00, overlap	31.0, CH	1.92–2.02, overlap	1.92–2.01, overlap
17	27.3, CH_2_	1.01, d (11.9) 1.66–1.71, m	27.3, CH_2_	1.02, d (11.8) 1.65–1.73, m	1.01, d (11.5) 1.65–1.69, m
18	30.2, CH_2_	1.57–1.60, m 1.82–1.87, m	30.3, CH_2_	1.56–1.60, m 1.84–1.91, m	1.55–1.59, m 1.84–1.88, m
19	68.0, CH	4.26, brs	69.6, CH	4.27, brs	4.25, brs
20	43.9, C		43.0, C		
21	21.5, CH_2_	1.61–1.65, overlap 2.16, dd (12.4, 7.7)	21.7, CH_2_	1.61–1.64, m 2.14, dd (12.3, 7.4)	1.59–1.62, m 2.12, dd (12.5, 7.3)
22	20.5, CH_2_	1.91–1.96, m 2.26–2.32, m	21.3, CH_2_	1.92–2.02, overlap 2.25–2.32, m	1.92–2.01, overlap 2.26–2.30, m
23	135.6, C		137.1, C		
24	35.3, CH	2.67–2.72, m	38.7, CH	2.50–2.53, m	2.51–2.54, m
25	15.3, CH_3_	0.77, d (7.2)	15.4, CH_3_	0.75, d (7.0)	0.74, d (7.1)
26	66.6, CH_2_	3.84, dd (10.3, 7.1) 3.93, dd (10.3, 9.9)	65.1, CH_2_	3.20–3.27, m 4.30, t (4.6)	3.20–3.23, m 4.27, t (5.0)
27	19.1, CH_3_	1.11, d (7.5)	19.3, CH_3_	1.10, d (7.2)	1.09, d (7.2)
28	19.3, CH_3_	0.93, d (6.5)	19.4, CH_3_	0.94, d (7.1)	0.93, d (6.8)
29	13.3, CH_3_	1.14, s	13.3, CH_3_	1.15, s	1.13, s
30	170.2, C				
31	20.8, CH_3_	1.98, s			
14-OH		4.08, d (4.5)		4.06, d (4.3)	4.04, d (4.5)
19-OH		4.27, d, (4.2)		4.22, d (4.6)	4.21, d (4.2)
NH		10.93, d (1.8)		10.89, d (1.5)	10.89, d (1.4)

**Table 2:** The results of *α*-glucosidase inhibitory activity (*n* = 3).

Compounds	1	2	3	4	5	6	Acarbose
IC_50_ ± SD (μM)	>300	>300	>300	>300	29.22 ± 0.83	>300	387.27 ± 19.02

## Data Availability

Data are contained within the article and Supplementary Materials.

## Supplementary Materials

The following supporting information can be downloaded at: https://www.mdpi.com/article/10.3390/molecules28237931/s1. Table S1: The X-ray single-crystal experimental details; Table S2: Geometric parameters (Å, °); Table S3: Hydrogen atom coordinates and isotropic displacement parameters; Table S4: ^1^H (600 MHz) and ^13^C (150 MHz) NMR data of compounds **3**–**6**; Figure S1: HRESIMS spectrum of compound **1**; Figure S2: ^1^H-NMR spectrum of compound **1** in DMSO-*d*_6_ (600 MHz); Figure S3: ^13^C-NMR spectrum of compound **1** in DMSO-*d*_6_ (150 MHz); Figure S4: HSQC spectrum of compound **1** in DMSO-*d*_6_ (600 × 150 MHz); Figure S5: HMBC spectrum of compound **1** in DMSO-*d*_6_ (600 × 150 MHz); Figure S6: ^1^H-^1^H COSY spectrum of compound **1** in DMSO-*d*_6_ (600 MHz); Figure S7: NOESY spectrum of compound **1** in DMSO-*d*_6_ (600 MHz); Figure S8: ^1^H-NMR spectrum of compound **2** in DMSO-*d*_6_ (150 MHz); Figure S9: ^13^C-NMR spectrum of compound **2** in DMSO-*d*_6_ (150 MHz); Figure S10: ^1^H-NMR spectrum of compound **3** in Chloroform-*d*_6_ (600 MHz); Figure S11: ^13^C-NMR spectrum of compound **3** in DMSO-*d*_6_ (150 MHz); Figure S12: ^1^H-NMR spectrum of compound **4** in Chloroform-*d*_3_ (600 MHz); Figure S13: ^13^C-NMR spectrum of compound **4** in DMSO-*d*_6_ (150 MHz); Figure S14: ^1^H-NMR spectrum of compound **5** in Chloroform-*d*_3_ (600 MHz); Figure S15: ^13^C-NMR spectrum of compound **5** in Chloroform-*d*_3_ (150 MHz); Figure S16: ^1^H-NMR spectrum of compound **6** in Chloroform-*d*_3_ (600 MHz); Figure S17: ^13^C-NMR spectrum of compound **6** in Chloroform-*d*_3_ (150 MHz); Figure S18: ^1^H-NMR spectrum of hydrolysate of compound **1** in DMSO-*d*_6_ (600 MHz).
