# Supplementary material for: Indole Diterpene Derivatives from the Aspergillus flavus GZWMJZ-288, an Endophytic Fungus from Garcinia multiflora
Source: Molecules. 2023 Dec 4;28(23):7931. doi: 10.3390/molecules28237931 (PMC10707737; doi:10.3390/molecules28237931)

*Supplementary Materials*

# Indole diterpene derivatives from the *Aspergillus flavus* GZWMJZ-288, an endophytic fungus from *Garcinia multiflora*

**Dongyang Wang [1,2,†], Xiaohong Zhuang [1,2,3,†], Ying Yin [1,2,3,†], Dan Wu [1,2], Wenwen He [1,2], Weiming Zhu [1,4], Yanchao Xu [1,3], Mingxing Zuo [1,2,*] and Liping Wang [1,2,3,*]**

1 State Key Laboratory of Functions and Applications of Medicinal Plants, Guizhou Medical University, Guiyang 550014, China; wangdongyang@gmc.edu.cn (Dongyang Wang)
2 Natural Products Research Center of Guizhou Province, Guiyang 550014, China
3 School of Pharmaceutical Sciences, Guizhou Medical University, Guiyang 550025, China
4 School of Medicine and Pharmacy, Ocean University of China, Qingdao 266003, China
* Correspondence: zuomingxing@gmc.edu.cn (M.Z.); wangliping2022@gmc.edu.cn (L.W.)
† These authors contributed equally to the work.

**Table S1.** The X-ray single crystal experimental details.

| Crystal data | Compound 2 (CCDC 2222865) |
| --- | --- |
| Chemical formula | $C_{28}H_{39}O_3N$ |
| $M_r$ | 437.60 |
| Crystal system, space group | Monoclinic, $P2_1$ |
| Temperature (K) | 150 |
| $a$, $b$, $c$ (Å) | 9.3756 (3), 10.4260 (4), 12.5667 (5) |
| α, β, γ (°) | 90, 96.673 (2), 90 |
| $V$ (Å$^3$) | 1220.07 (8) |
| $Z$ | 2 |
| Radiation | CuK$\alpha$ ($\lambda$ = 1.54178) |
| μ/mm$^{-1}$ | 0.595 |
| Crystal size (mm$^3$) | 0.2 × 0.15 × 0.1 |
| Data collection | |
| Diffractometer | Bruker D8 goniometer with CCD area detector |
| Absorption correction | Multi-scan *SADABS* |
| $T_{min}$, $T_{max}$ | 0.898, 0.942 |
| No. of measured, independent and observed [$I > 2\sigma(I)$] reflections | 10667, 4676, 4676 |
| $R_{int}$ | 0.0407 |
| Refinement | |
| $R[F^2 > 2\sigma(F^2)]$, $wR(F^2)$, $S$ | 0.0710, 0.1633 |
| No. of data | 4676 |
| No. of parameters | 296 |
| No. of restraints | 2 |
| Largest diff. peak/hole (e Å$^{-3}$) | 0.48, -0.21 |
| Index ranges | $-11 \le h \le 11$, $-12 \le k \le 12$, $-15 \le l \le 15$ |
| Flack parameter | 0.06 (13) |

A suitable crystal was selected and on a Bruker APEX-II CCD diffractometer. The crystal was kept at 150.00 K during data collection. Using Olex2, the structure was solved with the SHELXT structure solution program using Intrinsic Phasing and refined with the SHELXL refinement package using Least Squares minimisation.

**Table S2.** Geometric parameters (Å, º).

| Atom-Atom | Length/Å | Atom-Atom | Length/Å |
|---|---|---|---|
| O001-C00F | 1.431(4) | C00B-C00D | 1.592(4) |
| O002-C00K | 1.443(4) | C00B-C00K | 1.560(4) |
| O003-C00V | 1.426(4) | C00B-C00L | 1.575(4) |
| N004-C005 | 1.368(4) | C00B-C00U | 1.542(5) |
| N004-C00E | 1.367(5) | C00C-C00E | 1.369(5) |
| C005-C00I | 1.403(4) | C00C-C00I | 1.441(4) |
| C005-C00M | 1.398(5) | C00D-C00F | 1.557(4) |
| C006-C00A | 1.528(4) | C00D-C00H | 1.554(4) |
| C006-C00D | 1.561(4) | C00F-C00O | 1.525(4) |
| C006-C00J | 1.566(4) | C00G-C00H | 1.538(4) |
| C007-C008 | 1.522(4) | C00I-C00N | 1.410(5) |
| C007-C00V | 1.513(5) | C00J-C00S | 1.541(5) |
| C007-C00W | 1.527(5) | C00L-C00P | 1.543(4) |
| C008-C00A | 1.337(4) | C00L-C00Q | 1.534(4) |
| C008-C00G | 1.511(5) | C00M-C00R | 1.381(6) |
| C009-C00J | 1.529(5) | C00N-C00T | 1.379(5) |
| C009-C00K | 1.508(5) | C00O-C00Q | 1.513(4) |
| C00A-C00C | 1.489(4) | C00R-C00T | 1.408(6) |

| Atom-Atom-Atom | Length/Å | Atom-Atom-Atom | Length/Å |
|---|---|---|---|
| C00E-N004-C005 | 108.9(3) | C00H-C00D-C006 | 106.9(2) |
| N004-C005-C00I | 107.8(3) | C00H-C00D-C00B | 113.6(2) |
| N004-C005-C00M | 129.5(3) | C00H-C00D-C00F | 106.6(2) |
| C00M-C005-C00I | 122.7(3) | N004-C00E-C00C | 110.5(3) |
| C00A-C006-C00D | 112.4(2) | O001-C00F-C00D | 109.5(2) |
| C00A-C006-C00J | 109.9(2) | O001-C00F-C00O | 109.5(2) |
| C00D-C006-C00J | 117.0(3) | C00O-C00F-C00D | 114.3(2) |
| C008-C007-C00W | 111.3(3) | C008-C00G-C00H | 115.3(3) |
| C00V-C007-C008 | 111.3(3) | C00G-C00H-C00D | 111.9(2) |
| C00V-C007-C00W | 111.7(3) | C005-C00I-C00C | 107.3(3) |
| C00A-C008-C007 | 121.6(3) | C005-C00I-C00N | 118.8(3) |
| C00A-C008-C00G | 121.8(3) | C00N-C00I-C00C | 133.9(3) |
| C00G-C008-C007 | 116.5(3) | C009-C00J-C006 | 110.7(3) |
| C00K-C009-C00J | 111.6(3) | C009-C00J-C00S | 110.3(3) |
| C008-C00A-C006 | 122.9(3) | C00S-C00J-C006 | 116.6(3) |
| C008-C00A-C00C | 122.3(3) | O002-C00K-C009 | 111.3(2) |
| C00C-C00A-C006 | 114.7(3) | O002-C00K-C00B | 114.1(3) |
| C00K-C00B-C00D | 106.6(2) | C009-C00K-C00B | 113.3(3) |
| C00K-C00B-C00L | 111.9(2) | C00P-C00L-C00B | 115.2(3) |
| C00L-C00B-C00D | 109.2(2) | C00Q-C00L-C00B | 111.6(2) |
| C00U-C00B-C00D | 112.7(2) | C00Q-C00L-C00P | 108.2(3) |
| C00U-C00B-C00K | 107.2(3) | C00R-C00M-C005 | 117.2(3) |
| C00U-C00B-C00L | 109.3(3) | C00T-C00N-C00I | 118.9(3) |
| C00E-C00C-C00A | 127.5(3) | C00Q-C00O-C00F | 111.7(3) |
| C00E-C00C-C00I | 105.6(3) | C00O-C00Q-C00L | 110.9(3) |
| C00I-C00C-C00A | 126.8(3) | C00M-C00R-C00T | 121.4(3) |
| C006-C00D-C00B | 110.9(2) | C00N-C00T-C00R | 121.0(3) |
| C00F-C00D-C006 | 107.7(2) | O003-C00V-C007 | 109.7(3) |

| | |
|---|---|
| C00F-C00D-C00B | 111.0(2) |

| Atom-Atom-Atom-Atom | Length/Å | Atom-Atom-Atom-Atom | Length/Å |
|---|---|---|---|
| O001-C00F-C00O-C00Q | 70.1(3) | C00E-C00C-C00I-C00N | -178.7(3) |
| N004-C005-C00I-C00C | -0.4(3) | C00F-C00D-C00H-C00G | 55.0(3) |
| N004-C005-C00I-C00N | 178.6(3) | C00F-C00O-C00Q-C00L | 56.6(4) |
| N004-C005-C00M-C00R | -177.1(3) | C00G-C008-C00A-C006 | -1.2(5) |
| C005-N004-C00E-C00C | -0.6(4) | C00G-C008-C00A-C00C | -179.9(3) |
| C005-C00I-C00N-C00T | -0.9(5) | C00H-C00D-C00F-O001 | 51.5(3) |
| C005-C00M-C00R-C00T | -1.2(5) | C00H-C00D-C00F-C00O | 174.8(3) |
| C006-C00A-C00C-C00E | 92.7(4) | C00I-C005-C00M-C00R | 0.7(5) |
| C006-C00A-C00C-C00I | -84.4(4) | C00I-C00C-C00E-N004 | 0.3(3) |
| C006-C00D-C00F-O001 | 165.9(2) | C00I-C00N-C00T-C00R | 0.4(5) |
| C006-C00D-C00F-C00O | -70.9(3) | C00J-C006-C00A-C008 | 109.2(3) |
| C006-C00D-C00H-C00G | -59.9(3) | C00J-C006-C00A-C00C | -72.0(3) |
| C007-C008-C00A-C006 | 175.0(3) | C00J-C006-C00D-C00B | 47.5(3) |
| C007-C008-C00A-C00C | -3.7(5) | C00J-C006-C00D-C00F | 169.1(2) |
| C007-C008-C00G-C00H | 177.2(3) | C00J-C006-C00D-C00H | -76.8(3) |
| C008-C007-C00V-O003 | -166.3(3) | C00J-C009-C00K-O002 | 167.1(2) |
| C008-C00A-C00C-C00E | -88.5(4) | C00J-C009-C00K-C00B | -62.8(3) |
| C008-C00A-C00C-C00I | 94.4(4) | C00K-C009-C00J-C006 | 50.3(4) |
| C008-C00G-C00H-C00D | 38.2(4) | C00K-C009-C00J-C00S | -80.3(3) |
| C00A-C006-C00D-C00B | 176.0(2) | C00K-C00B-C00D-C006 | -52.5(3) |
| C00A-C006-C00D-C00F | -62.4(3) | C00K-C00B-C00D-C00F | -172.1(2) |
| C00A-C006-C00D-C00H | 51.8(3) | C00K-C00B-C00D-C00H | 67.8(3) |
| C00A-C006-C00J-C009 | -174.8(2) | C00K-C00B-C00L-C00P | -61.7(4) |
| C00A-C006-C00J-C00S | -47.6(4) | C00K-C00B-C00L-C00Q | 174.4(3) |
| C00A-C008-C00G-C00H | -6.5(4) | C00L-C00B-C00D-C006 | 68.5(3) |
| C00A-C00C-C00E-N004 | -177.2(3) | C00L-C00B-C00D-C00F | -51.1(3) |
| C00A-C00C-C00I-C005 | 177.7(3) | C00L-C00B-C00D-C00H | -171.1(3) |
| C00A-C00C-C00I-C00N | -1.2(5) | C00L-C00B-C00K-O002 | 71.8(3) |
| C00B-C00D-C00F-O001 | -72.6(3) | C00L-C00B-C00K-C009 | -56.9(3) |
| C00B-C00D-C00F-C00O | 50.7(3) | C00M-C005-C00I-C00C | -178.7(3) |
| C00B-C00D-C00H-C00G | 177.5(3) | C00M-C005-C00I-C00N | 0.4(5) |
| C00B-C00L-C00Q-C00O | -59.8(4) | C00M-C00R-C00T-C00N | 0.7(6) |
| C00C-C00I-C00N-C00T | 177.8(3) | C00P-C00L-C00Q-C00O | 172.4(3) |
| C00D-C006-C00A-C008 | -23.0(4) | C00U-C00B-C00D-C006 | -169.8(3) |
| C00D-C006-C00A-C00C | 155.8(3) | C00U-C00B-C00D-C00F | 70.5(3) |
| C00D-C006-C00J-C009 | -45.1(4) | C00U-C00B-C00D-C00H | -49.5(3) |
| C00D-C006-C00J-C00S | 82.1(4) | C00U-C00B-C00K-O002 | -48.0(3) |
| C00D-C00B-C00K-O002 | -168.9(2) | C00U-C00B-C00K-C009 | -176.7(3) |
| C00D-C00B-C00K-C009 | 62.4(3) | C00U-C00B-C00L-C00P | 56.8(4) |
| C00D-C00B-C00L-C00P | -179.5(3) | C00U-C00B-C00L-C00Q | -67.1(3) |
| C00D-C00B-C00L-C00Q | 56.6(4) | C00V-C007-C008-C00A | 121.8(4) |
| C00D-C00F-C00O-C00Q | -53.2(4) | C00V-C007-C008-C00G | -61.8(4) |
| C00E-N004-C005-C00I | 0.6(3) | C00W-C007-C008-C00A | -112.8(4) |
| C00E-N004-C005-C00M | 178.7(3) | C00W-C007-C008-C00G | 63.6(4) |
| C00E-C00C-C00I-C005 | 0.1(3) | C00W-C007-C00V-O003 | 68.6(4) |

**Table S3.** Hydrogen Atom Coordinates and Isotropic Displacement Parameters.

| Atom | *x* | *y* | *z* | U(eq) |
|---|---|---|---|---|
| H001 | 1358.98 | 1335.02 | 7097.79 | 76 |
| H002 | 8280.04 | 2819.32 | 8887.46 | 79 |
| H003 | 938.16 | 6788.82 | 2766.66 | 85 |
| H004 | 2822.48 | 9601.31 | 7184.8 | 59 |
| H006 | 3843.03 | 4849.67 | 8149.19 | 51 |
| H007 | 1049.32 | 6340.24 | 5331.41 | 57 |
| H00A | 7736.55 | 4873.08 | 7952.94 | 59 |
| H00B | 6661 | 4674.86 | 8840.37 | 59 |
| H00E | 3918.81 | 7885.66 | 6266.57 | 59 |
| H00F | 1780.83 | 3298.36 | 7176.58 | 53 |
| H00C | 3036.38 | 3522.93 | 4648.09 | 59 |
| H00D | 1941.51 | 2914.46 | 5390.49 | 59 |
| H00G | 4968.69 | 3169.02 | 5870.12 | 55 |
| H00H | 4016.4 | 1915.08 | 5994.76 | 55 |
| H00J | 5569.8 | 6151.47 | 7720.81 | 57 |
| H00K | 6717.67 | 3055.7 | 7035.26 | 57 |
| H00L | 4993.69 | 3635.36 | 9404.64 | 57 |
| H00M | 734.03 | 9855.15 | 8551.97 | 65 |
| H00N | 550.15 | 5336.19 | 8160.62 | 61 |
| H00I | 2325.91 | 3779.17 | 8971.88 | 58 |
| H00O | 1391.84 | 2489.75 | 8898.81 | 58 |
| H00P | 6315.27 | 1205.56 | 9913.94 | 91 |
| H00Q | 5781.99 | 2111.85 | 10816.5 | 91 |
| H00R | 7026.1 | 2588.92 | 10140 | 91 |
| H00S | 3464.1 | 1248.45 | 9325.6 | 62 |
| H00T | 3382 | 2311.22 | 10237.5 | 62 |
| H00U | -781.47 | 8611.11 | 9490.85 | 71 |
| H00V | 6875.97 | 6143.85 | 6314.71 | 86 |
| H00W | 5269.09 | 5871.42 | 5784.48 | 86 |
| H00X | 6396.2 | 4717.13 | 5966.15 | 86 |
| H00Y | -887.19 | 6381.52 | 9281.24 | 70 |
| H00Z | 4570.44 | 594.89 | 8082.51 | 75 |
| H | 6284.12 | 710.47 | 8228.39 | 75 |
| HA | 5402.52 | 908.94 | 7071.29 | 75 |
| H00 | 2579.76 | 6016.79 | 3937.12 | 75 |
| HB | 1558.07 | 4846.88 | 3511.42 | 75 |
| H1 | -189.15 | 3956.36 | 4592.16 | 106 |
| HC | -1019.02 | 5288.07 | 4671.37 | 106 |
| HD | -399.93 | 4543.89 | 5739.97 | 106 |

**Table S4.** $^{1}H$ (600 MHz) and $^{13}C$ (150 MHz) NMR data of compounds **3**-**6**.

| Position | 3 | | 4 | | 5 | | 6 | |
|---|---|---|---|---|---|---|---|---|
| | $\delta_C$ | $\delta_H$, (*J* in Hz) | $\delta_C$ | $\delta_H$, (*J* in Hz) | $\delta_C$ | $\delta_H$, (*J* in Hz) | $\delta_C$ | $\delta_H$, (*J* in Hz) |
| 2 | 122.0 | 6.91, d, (2.3) | 123.5 | 7.06, d, (2.2) | 138.5 | | 121.4 | 6.89, d, (1.8) |
| 3 | 116.6 | | 118.3 | | 118.8 | | 119.5 | |

| | | | | | | | | |
|---|---|---|---|---|---|---|---|---|
| 4 | 127.0 | | 127.0 | | 123.2 | | 127.7 | |
| 5 | 118.4 | 7.41, d, (8.0) | 117.4 | 7.35, d, (8.1) | 121.8 | 8.03, d, (7.9) | 119.7 | 7.44, d, (8.2) |
| 6 | 118.5 | 7.09, td, (7.5, 0.9) | 114.6 | 7.19, td, (7.4, 08) | 119.6 | 7.29, td, (7.9, 1.1) | 118.7 | 7.09, td, (7.5, 0.6) |
| 7 | 120.8 | 7.19, td, (7.2, 0.8) | 120.6 | 7.12, td, (7.5, 0.9) | 124.5 | 7.40, td, (7.2, 0.8) | 121.9 | 7.19, dt, (7.0, 0.7) |
| 8 | 111.5 | 7.38, d, (7.8) | 111.5 | 7.53, d, (7.8) | 110.8 | 7.42, d, (7.5) | 111.2 | 7.37, d, (8.2) |
| 9 | 135.9 | | 135.9 | | 139.6 | | 136.0 | |
| 10 | 125.5 | | 33.9 | 3.66, dd, (12.5, 5.5) | 34.7 | | 125.6 | |
| 11 | 43.1 | 2.48, d, (6.2) | 38.4 | 2.68, ddd, (5.2, 5.2, 0.8) | 38.9 | 4.22, dd, (6.4, 1.3) | 43.6 | 2.44, d, (4.8) |
| 12 | 29.8 | 1.50-1.52, m | 27.2 | 1.61-1.66, m | 31.5 | 2.86-2.91, m | 31.4 | 1.95-1.99, m |
| 13 | 35.4 | 0.84-0.88, m<br>1.72-1.84, m | 37.3 | 1.21, dddd, (12.5, 2.8, 2.8, 0.8)<br>1.74-1.83, m | 35.4 | 2.40-2.43, m<br>1.73, dd, (12.4, 1.8) | 25.8 | 1.63-1.65, m<br>1.15-1.18, m |
| 14 | 69.4 | 4.48-4.81, m | 69.5 | 4.66-4.69, m | 72.3 | 4.26, dd, (12.8, 3.2) | 27.7 | 1.55-1.58, m<br>1.19-1.22, m |
| 15 | 42.9 | | 42.6 | | 43.8 | | 38.6 | |
| 16 | 30.2 | 2.21-2.25, m | 30.6 | 2.19-2.24, m | 31.4 | 2.61-2.66, m | 29.2 | 2.03-2.08, m |
| 17 | 29.3 | 1.17-1.20, m<br>1.72-1.84, m | 30.0 | 1.74-1.83, m<br>1.33-1.34, overlap | 27.3 | 1.27-1.30, m<br>1.78-1.84, m | 25.5 | 1.25-1.27, m<br>1.65-1.68, m |
| 18 | 31.0 | 1.24-1.26, m<br>1.72-1.84, m | 31.0 | 1.74-1.83, m<br>1.33-1.34, overlap | 24.5 | 1.32-1.39, m<br>1.87-1.93, m | 21.9 | 1.74-1.78, m<br>2.03-2.08, m |
| 19 | 68.0 | 4.04, dd, (12.4, 3.5) | 66.1 | 3.98, dd, (12.3, 2.7) | 71.0 | 3.88, dd, (2.6, 2.6) | 71.3 | 4.49, bs |
| 20 | 44.0 | | 45.4 | | 44.0 | | 42.5 | |
| 21 | 21.6 | 1.88-1.92, m<br>2.12-2.25, m | 24.3 | 1.84-1.92, m<br>2.11-2.15, m | 22.0 | 2.16-2.22, m<br>2.74-2.81, m | 30.2 | 1.82-1.85, m<br>1.72-1.75, m |
| 22 | 27.3 | 1.88-1.92, m<br>2.12-2.25, m | 27.3 | 1.84-1.92, m<br>2.20-2.29, m | 30.3 | 2.44-2.47, m<br>2.91-2.95, m | 20.6 | 2.21-2.24, m<br>2.10-2.13, m |
| 23 | 139.7 | | 43.7 | 3.14-3.21, m | 126.0 | | 141.2 | |
| 24 | 30.4 | 2.56-2.61, m | 149.9 | | 135.1 | | 31.1 | 2.56-2.64, m |
| 25 | 21.7 | 1.01, d, (6.6) | 111.1 | 4.85, dd, (2.7, 2.7)<br>4.80, d, (1.8) | 109.3 | 7.08, s | 22.0 | 0.83, d, (6.9) |
| 26 | 20.0 | 0.95, d, (7.3) | 22.4 | 1.51, s | 20.7 | 2.38, s | 20.9 | 0.97, d, (6.8) |
| 27 | 19.2 | 0.83, d, (6.8) | 19.4 | 1.30, d, (7.1) | 19.2 | 0.67, d, (7.5) | 18.2 | 1.09, d, (7.5) |
| 28 | 19.4 | 1.15, d, (7.3) | 18.2 | 1.05, d, (6.8) | 19.6 | 1.25, d, (6.7) | 15.9 | 0.76, d, (6.8) |
| 29 | 13.3 | 1.28, s | 13.5 | 1.27, s | 13.2 | 1.41, s | 18.2 | 1.00, s |
| NH | | 8.08, brs | | 7.95, s | | 8.06, s | | 8.05, brs |

$^{1}$H (600 MHz) and $^{13}$C (150 MHz) NMR data of **5**, **6** in Chloroform-*d*$_3$, and $^{1}$HNMR **3**, **4** in Chloroform-*d*$_3$, $^{13}$CNMR in DMSO-*d*$_6$.

**Figure S1.** HRESIMS spectrum of compound **1.**

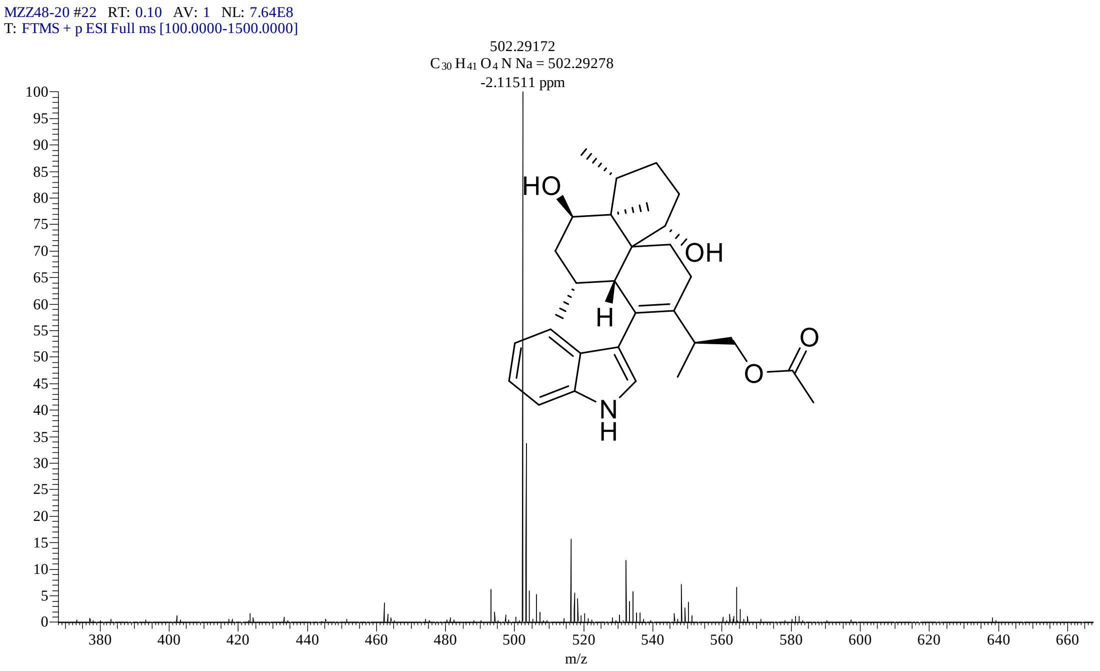


**Figure S2.** $^{1}$H-NMR spectrum of compound **1** in DMSO-d6 (600 MHz).

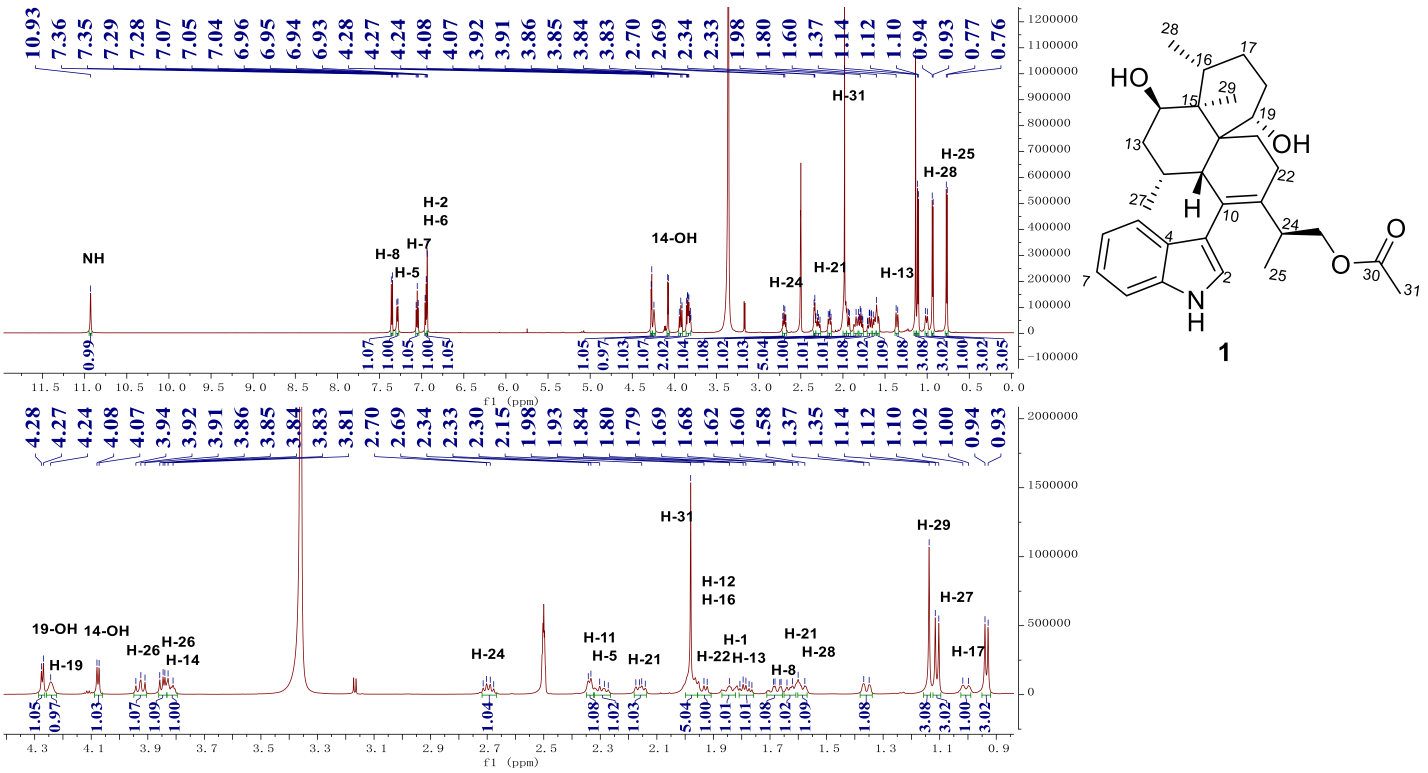

**Figure S3.** $^{13}$C-NMR spectrum of compound **1** in DMSO-$d_6$ (150 MHz).

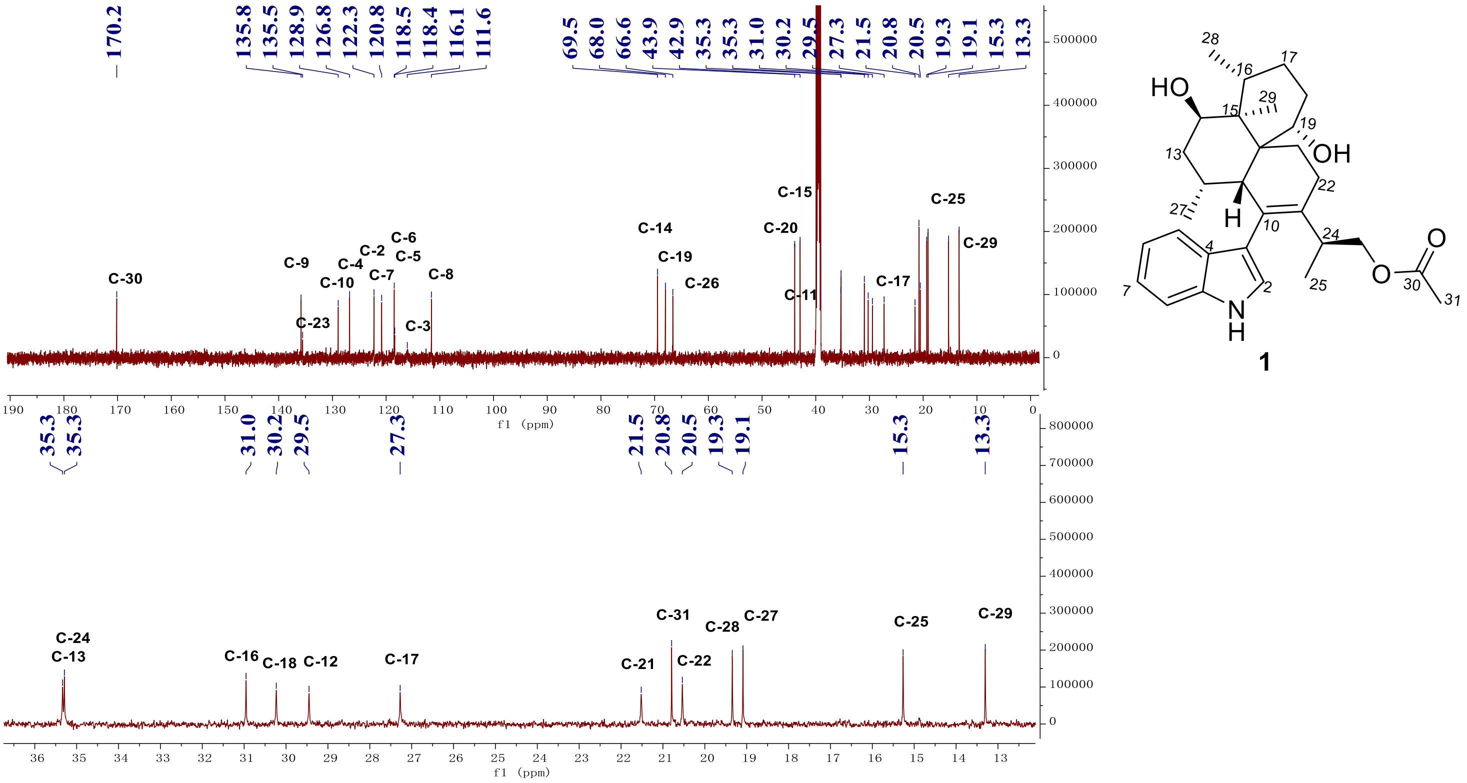

**Figure S4.** HSQC spectrum of compound **1** in DMSO-$d_6$ (600*150 MHz).

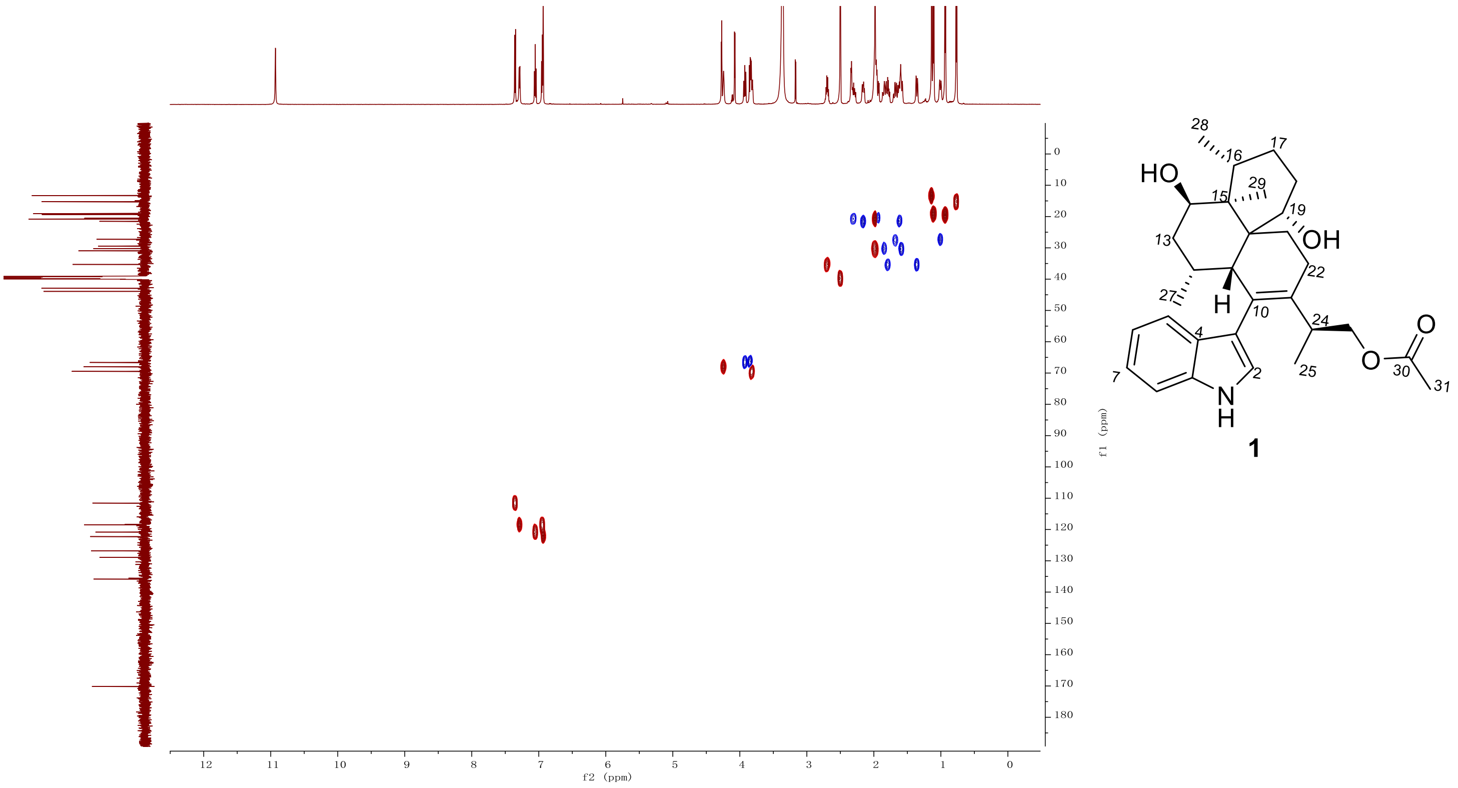

**Figure S5.** HMBC spectrum of compound **1** in DMSO-$d_6$ (600*150 MHz).

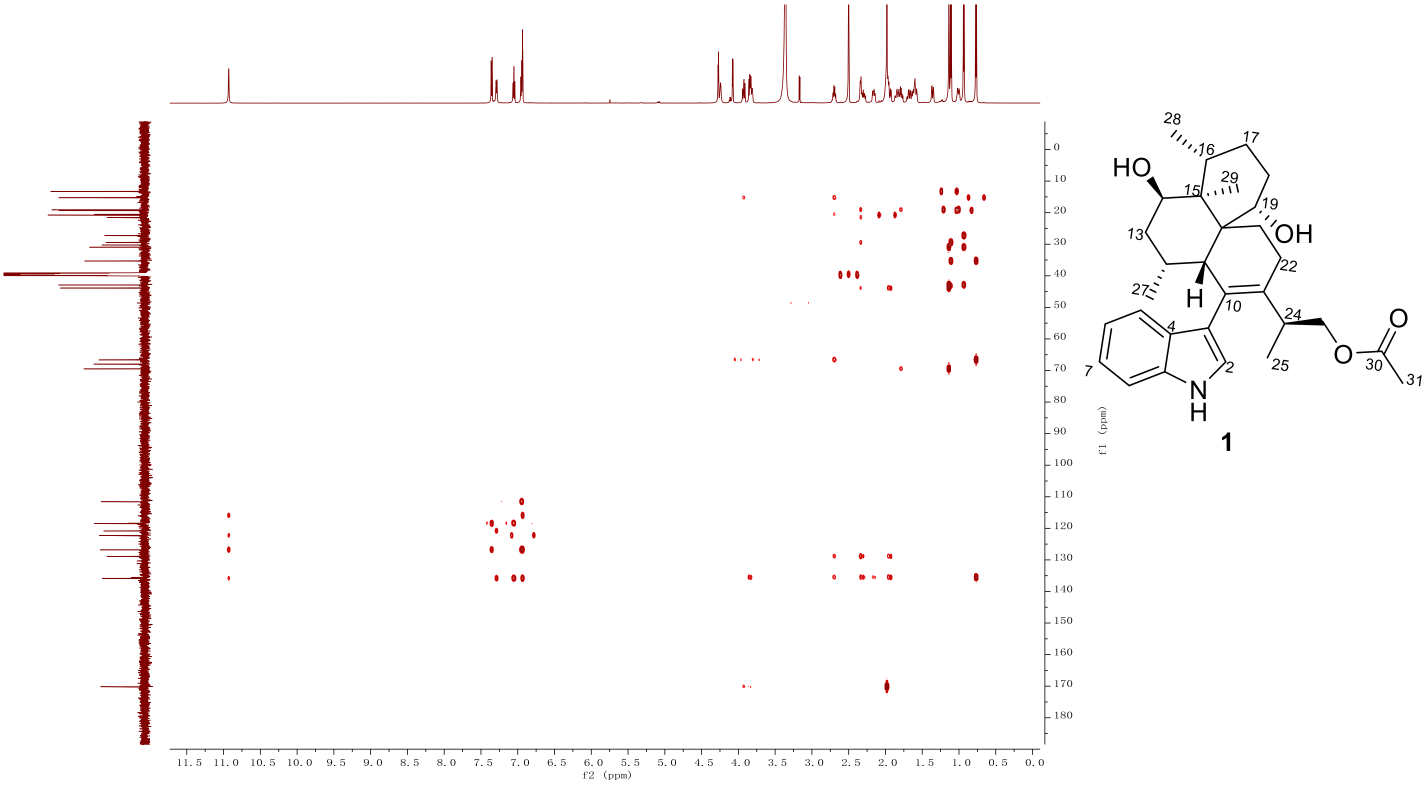


**Figure S6.** $^1$H-$^1$H COSY spectrum of compound **1** in DMSO-$d_6$ (600 MHz).

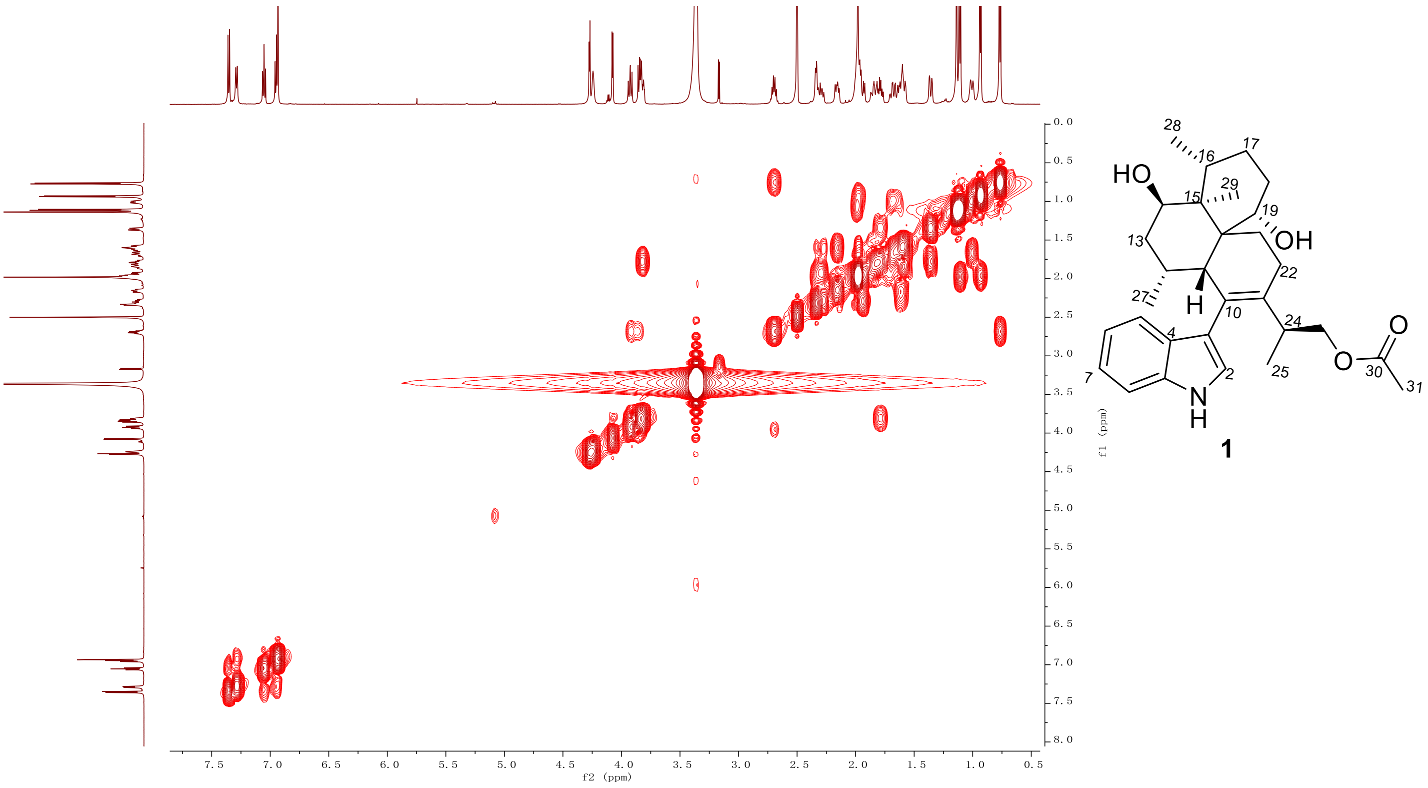

**Figure S7.** NOESY spectrum of compound **1** in DMSO-$d_6$ (600 MHz).

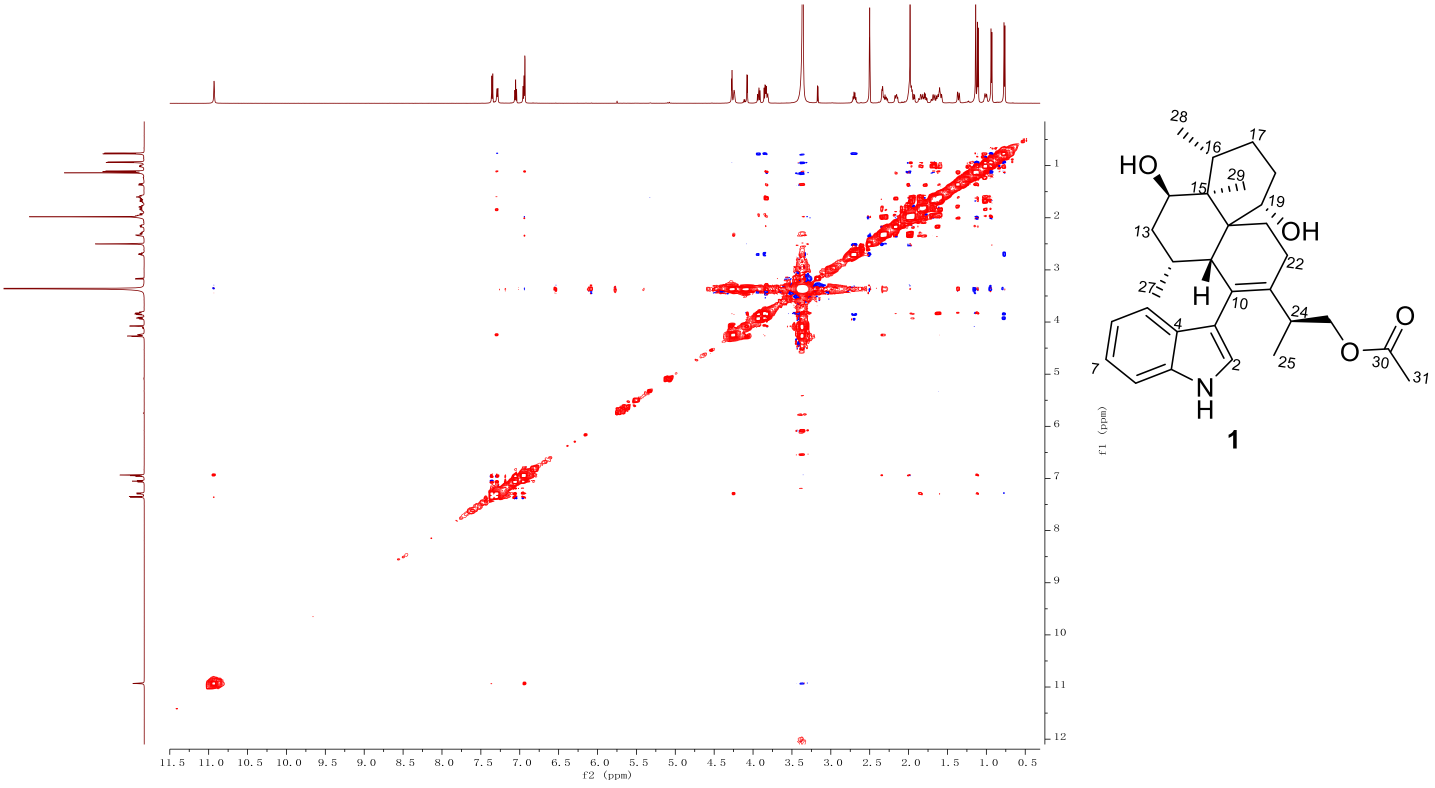


**Figure S8.** $^{1}$H-NMR spectrum of compound **2** in DMSO-$d_6$ (600 MHz).

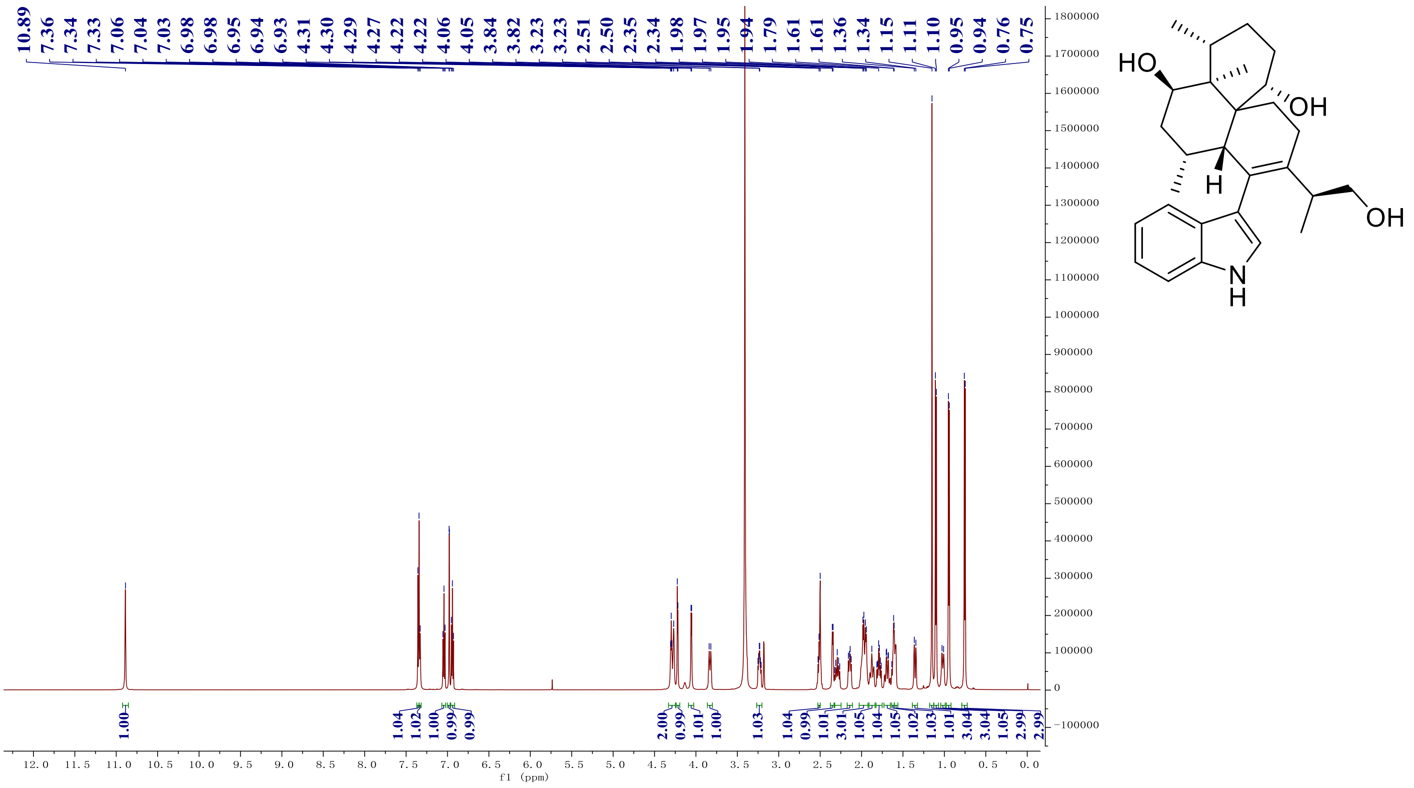

**Figure S9.** $^{13}$C-NMR spectrum of compound **2** in DMSO-$d_6$ (150 MHz).

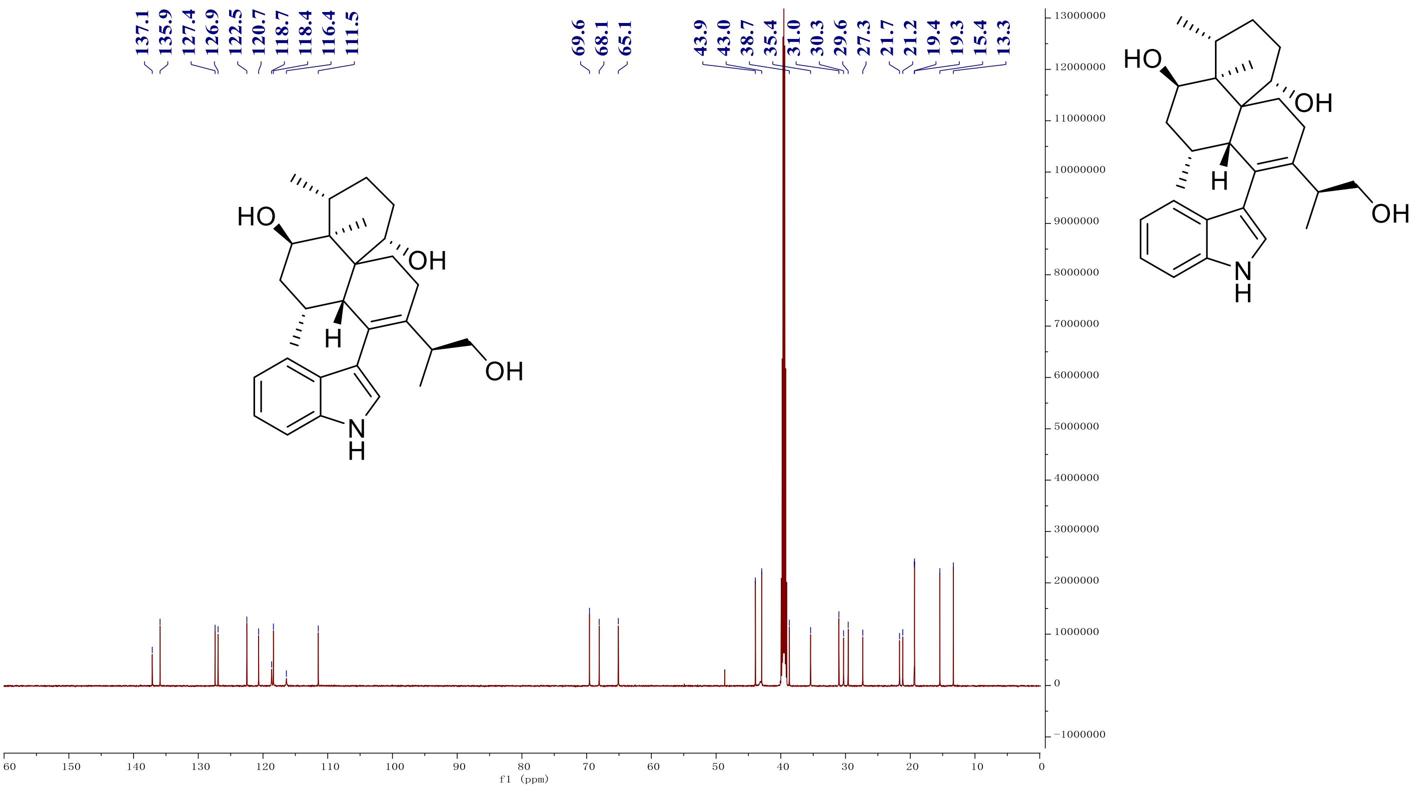


**Figure S10.** $^{1}$H-NMR spectrum of compound **3** in Chloroform-$d_3$ (600 MHz).

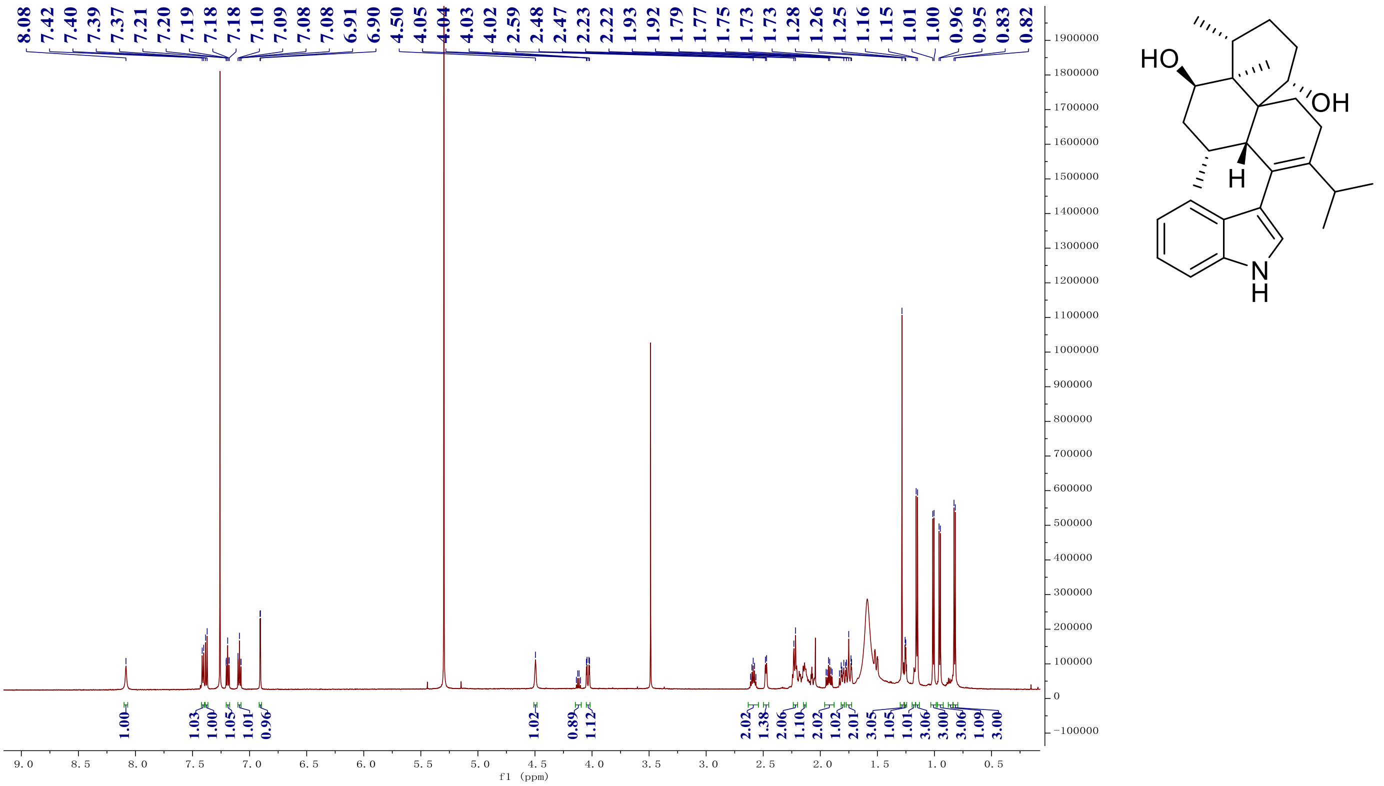

**Figure S11.** $^{13}$C-NMR spectrum of compound **3** in DMSO-$d_6$ (150 MHz).

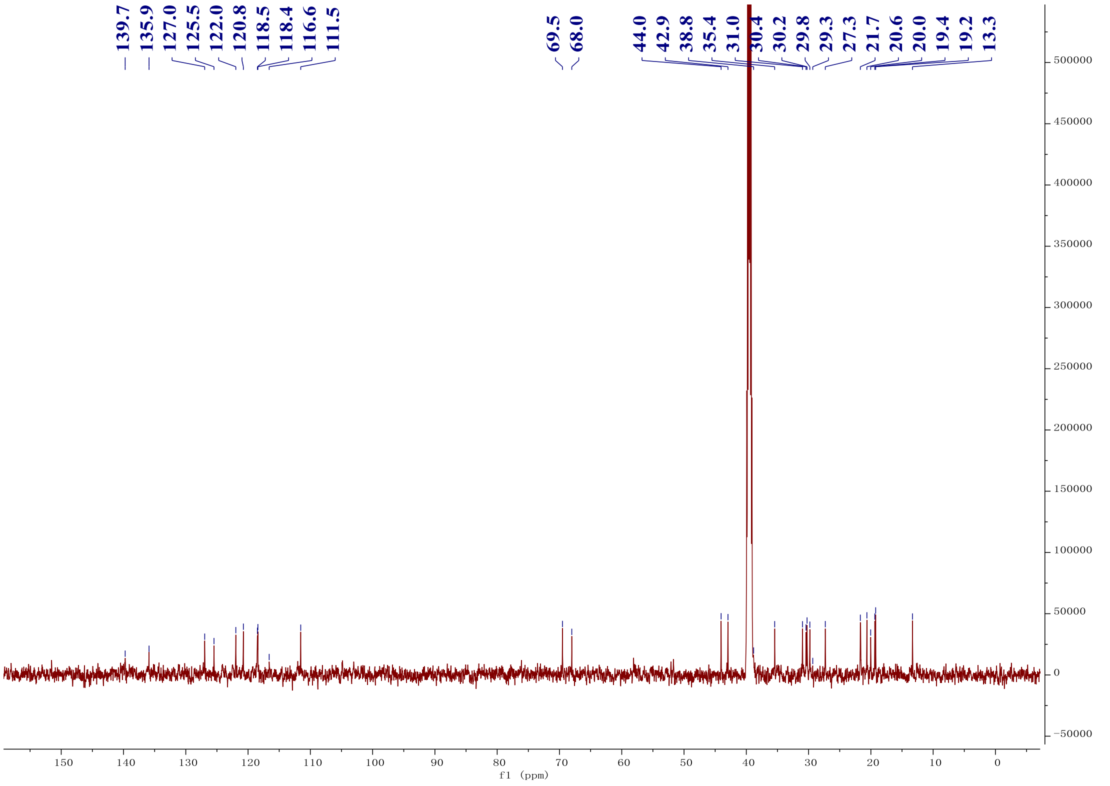


**Figure S12.** $^{1}$H-NMR spectrum of compound **4** in Chloroform-$d_3$ (600 MHz).

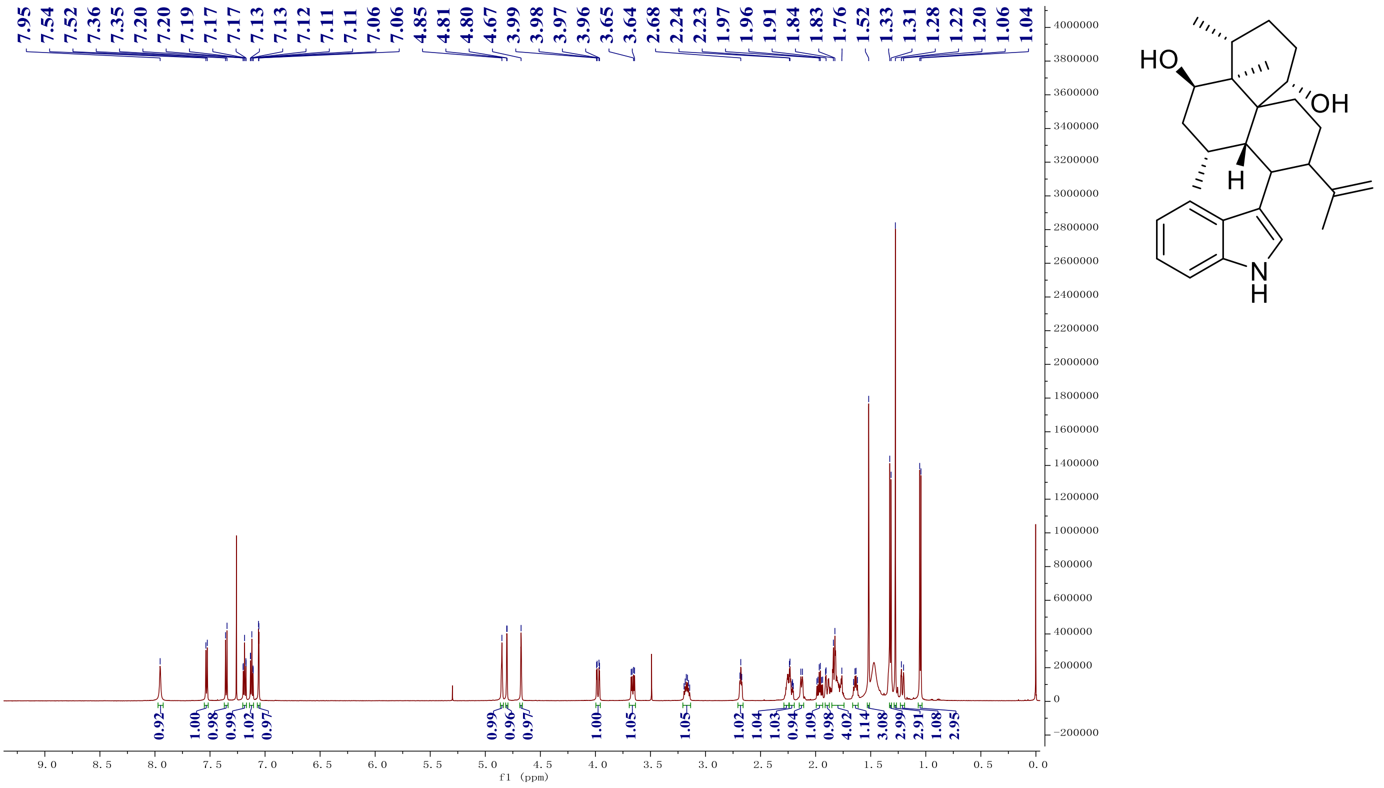

**Figure S13.** $^{13}$C-NMR spectrum of compound **4** in DMSO-$d_6$ (150 MHz).

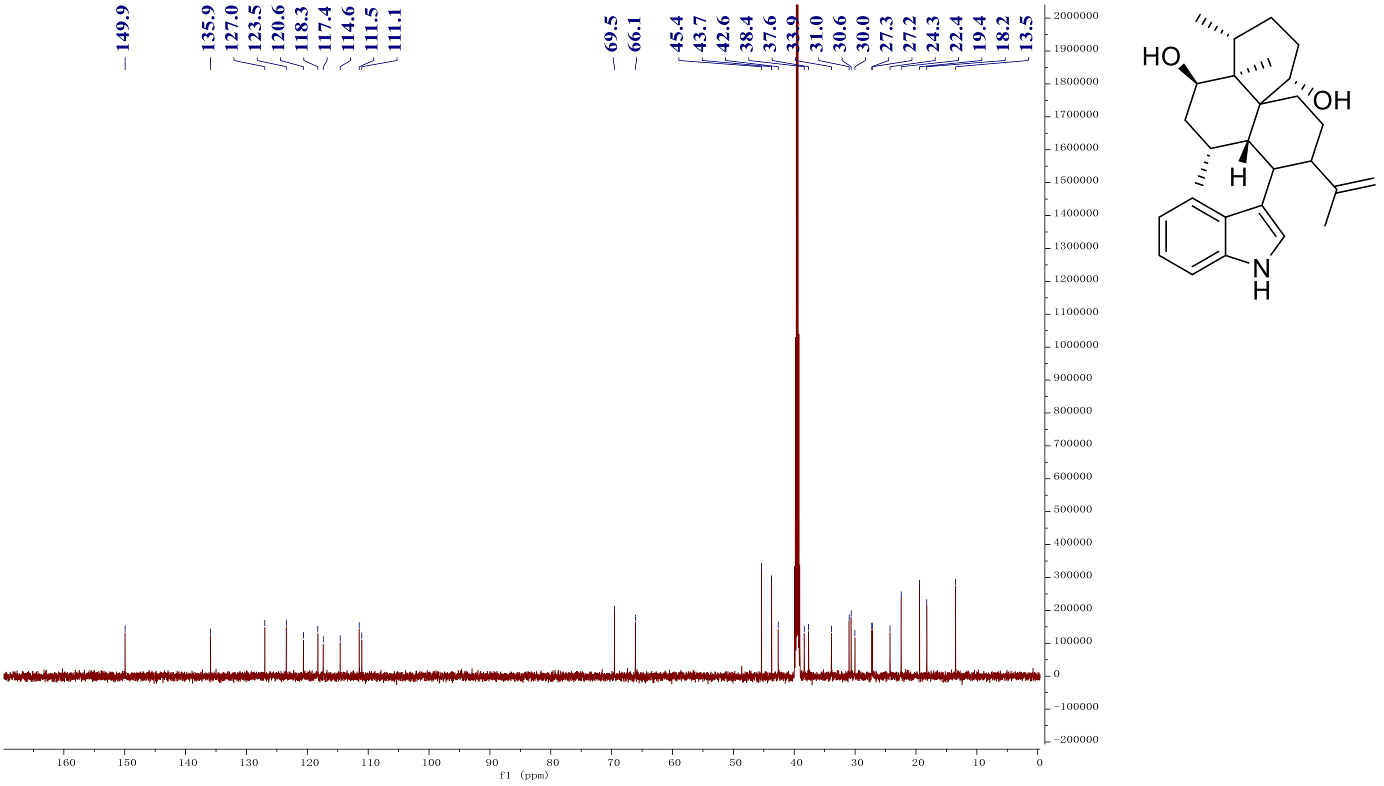


**Figure S14.** $^1$H-NMR spectrum of compound **5** in Chloroform-$d_3$ (600 MHz).

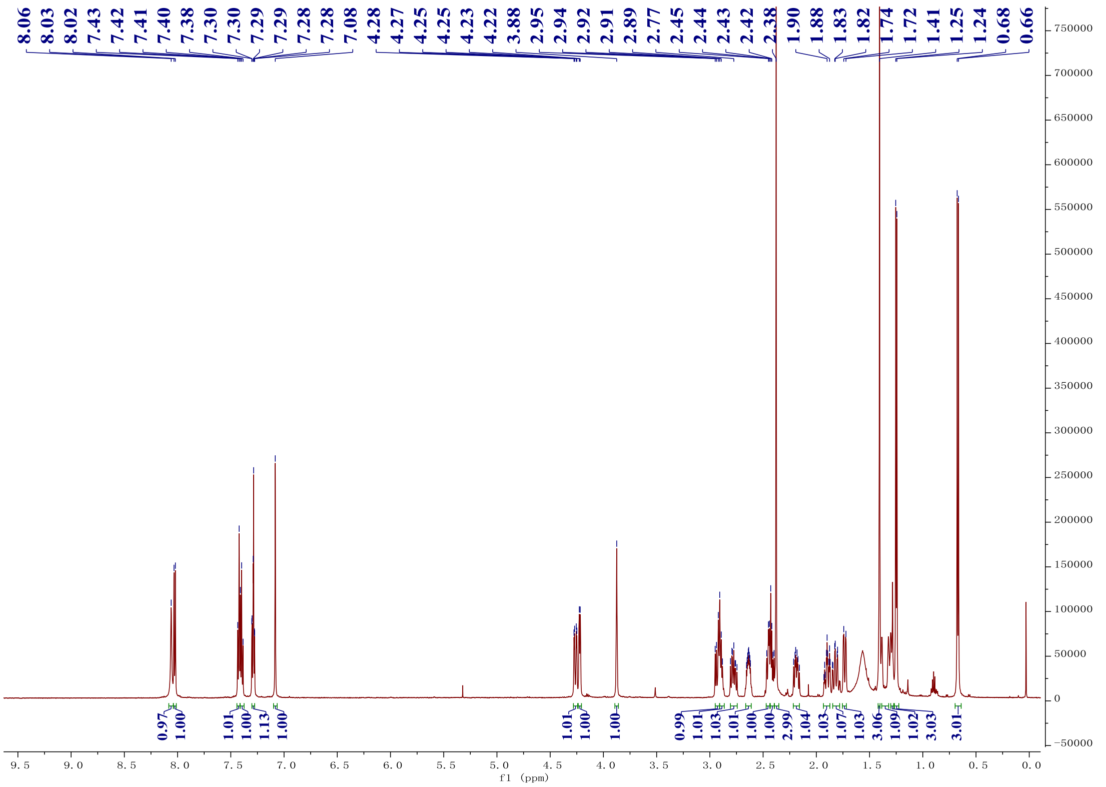

**Figure S15.** $^{13}$C-NMR spectrum of compound **5** in Chloroform-$d_3$ (150 MHz).

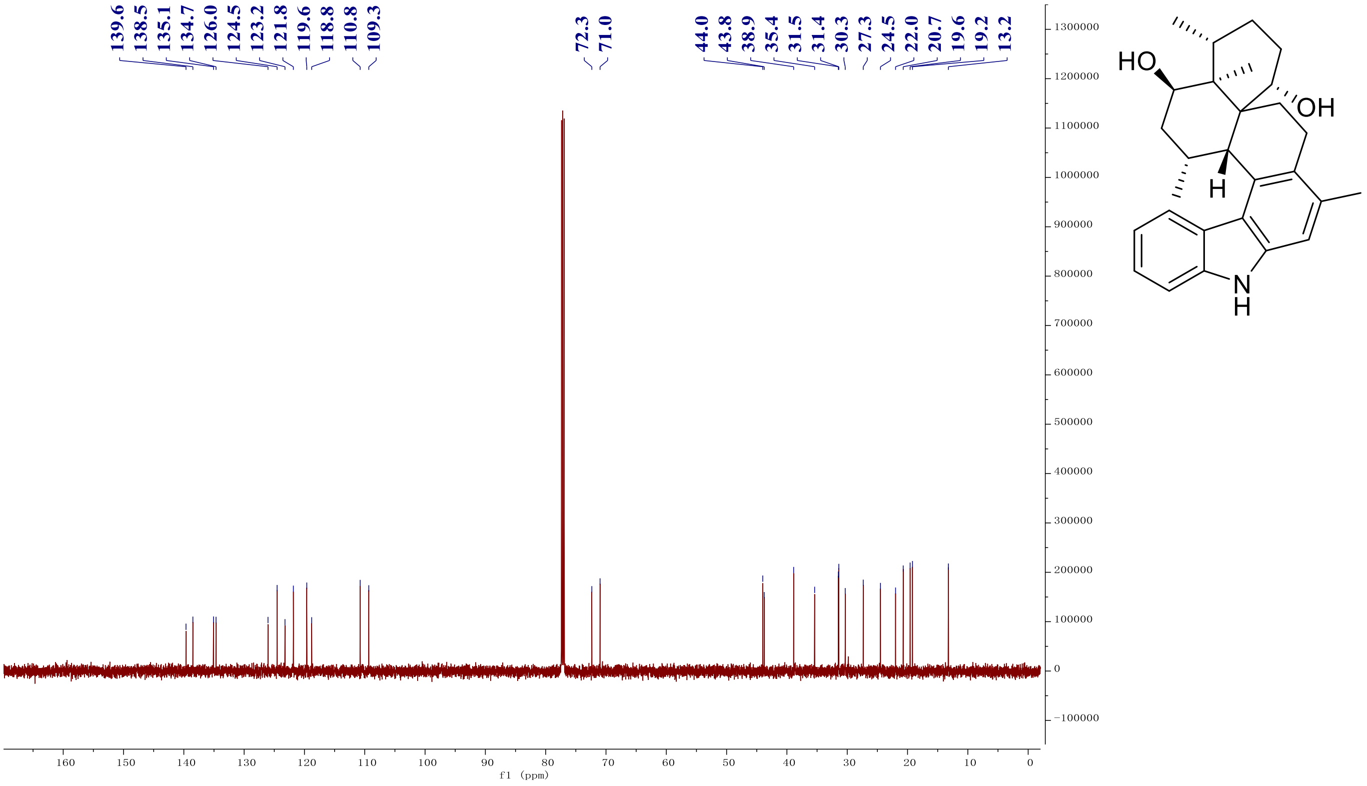


**Figure S16.** $^{1}$H-NMR spectrum of compound **6** in Chloroform-$d_3$ (600 MHz).

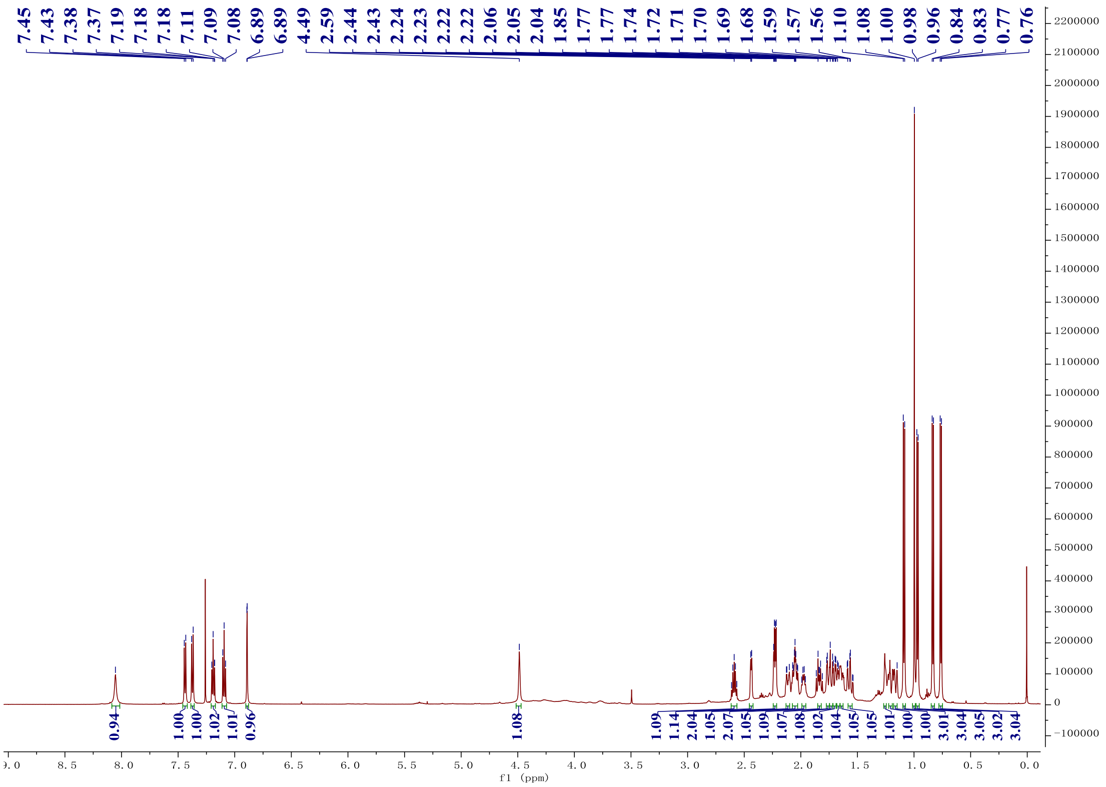

**Figure S17.** $^{13}$C-NMR spectrum of compound **6** in Chloroform-$d_3$ (150 MHz).

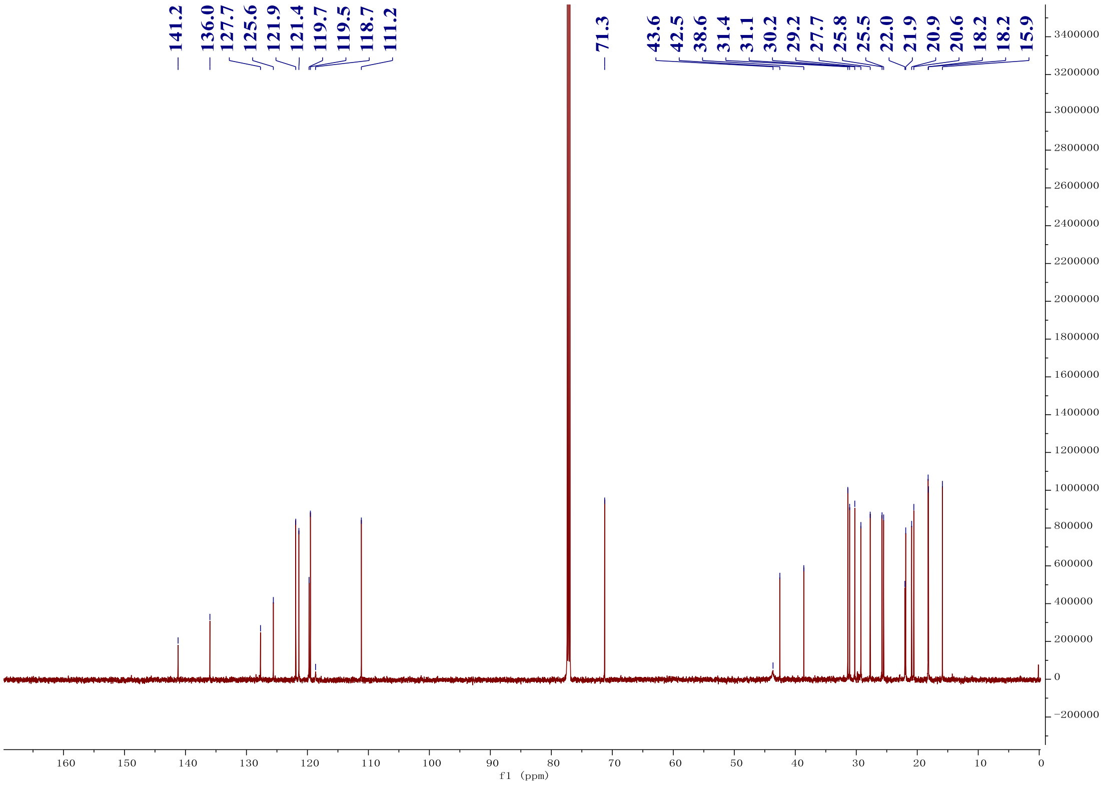


**Figure S18.** $^{1}$H-NMR spectrum of hydrolysate of compound **1** in DMSO-$d_6$ (600 MHz).

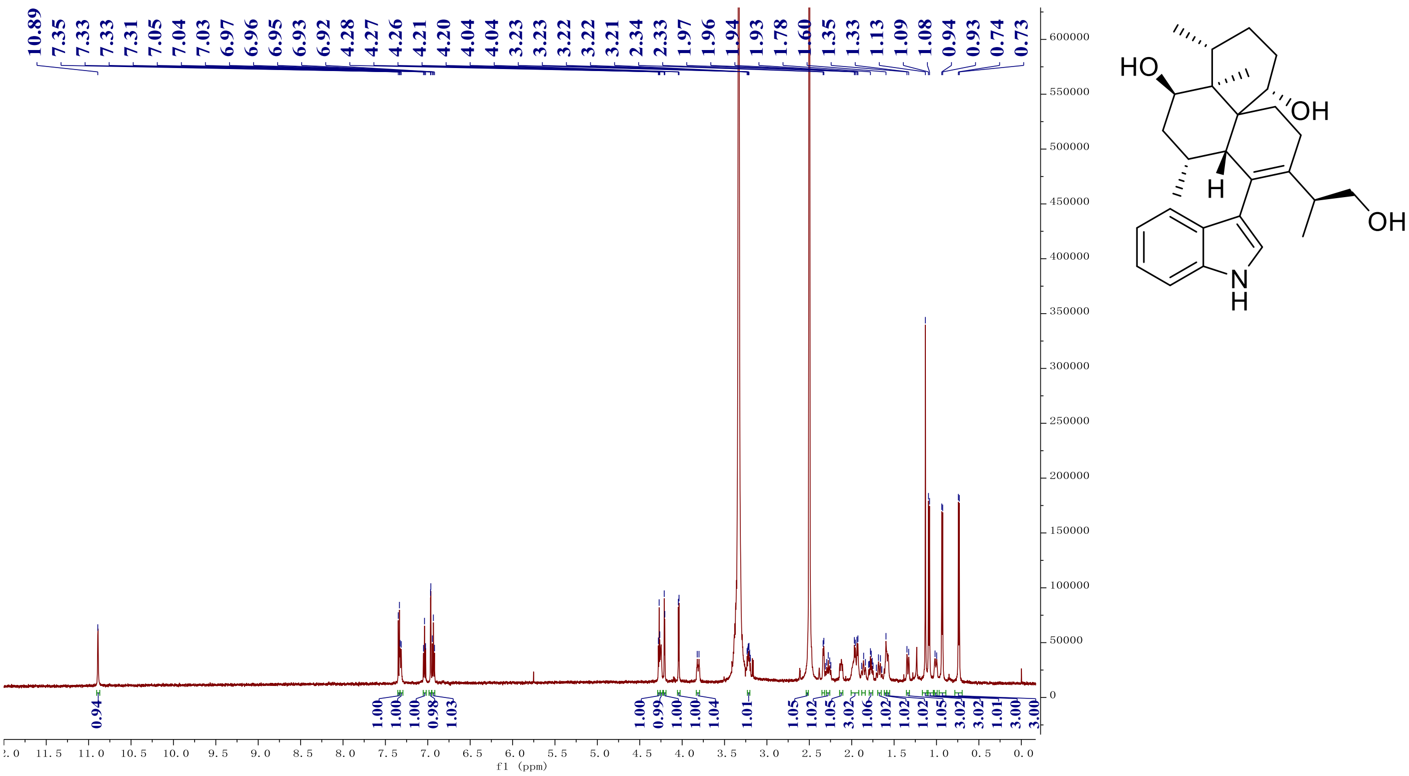

Supplement: Supplementary file 1 [file molecules-28-07931-s001.zip › molecules-2700014-supplementary.pdf]
